# Structural dynamics of therapeutic nucleic acids with phosphorothioate backbone modifications

**DOI:** 10.1093/nargab/lqae058

**Published:** 2024-05-25

**Authors:** Antonio Carlesso, Johanna Hörberg, Giuseppe Deganutti, Anna Reymer, Pär Matsson

**Affiliations:** Department of Pharmacology, Sahlgrenska Academy, University of Gothenburg, Box 431, SE-405 30 Gothenburg, Sweden; Department of Chemistry and Molecular Biology, University of Gothenburg, Box 462, SE-405 30 Gothenburg, Sweden; Centre for Health and Life Sciences, Coventry University, Coventry, UK; Department of Chemistry and Molecular Biology, University of Gothenburg, Box 462, SE-405 30 Gothenburg, Sweden; Department of Pharmacology, Sahlgrenska Academy, University of Gothenburg, Box 431, SE-405 30 Gothenburg, Sweden; SciLifeLab, University of Gothenburg, Sweden

## Abstract

Antisense oligonucleotides (ASOs) offer ground-breaking possibilities for selective pharmacological intervention for any gene product-related disease. Therapeutic ASOs contain extensive chemical modifications that improve stability to enzymatic cleavage and modulate binding affinity relative to natural RNA/DNA. Molecular dynamics (MD) simulation can provide valuable insights into how such modifications affect ASO conformational sampling and target binding. However, force field parameters for chemically modified nucleic acids (NAs) are still underdeveloped. To bridge this gap, we developed parameters to allow simulations of ASOs with the widely applied phosphorothioate (PS) backbone modification, and validated these in extensive all-atom MD simulations of relevant PS-modified NA systems representing B-DNA, RNA, and DNA/RNA hybrid duplex structures. Compared to the corresponding natural NAs, single PS substitutions had marginal effects on the ordered DNA/RNA duplex, whereas substantial effects of phosphorothioation were observed in single-stranded RNA and B-DNA, corroborated by the experimentally derived structure data. We find that PS-modified NAs shift between high and low twist states, which could affect target recognition and protein interactions for phosphorothioated oligonucleotides. Furthermore, conformational sampling was markedly altered in the PS-modified ssRNA system compared to that of the natural oligonucleotide, indicating sequence-dependent effects on conformational preference that may in turn influence duplex formation.

## Graphical Abstract

**Graphical Abstract:**
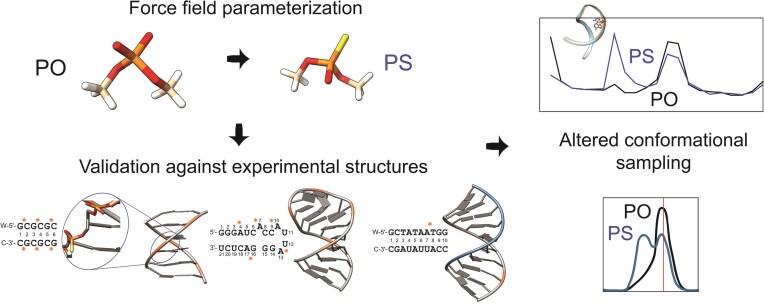

## Introduction

Antisense oligonucleotides (ASOs) open vast new opportunities in drug discovery by allowing selective modulation of, in principle, any disease-related gene (1,2). ASOs bind their cognate mRNA transcripts through Watson–Crick–Franklin base-pairing, resulting in either the degradation of a transcript and consequent knock-down of target protein expression, or inducing alternative gene splicing by sterically blocking the cellular mRNA splicing machinery. Unmodified oligonucleotides are rapidly hydrolyzed by cellular nucleases, and therapeutic ASOs are therefore chemically modified to improve metabolic stability while maintaining the desirable sequence-specific hybridization.

**Figure 1.**
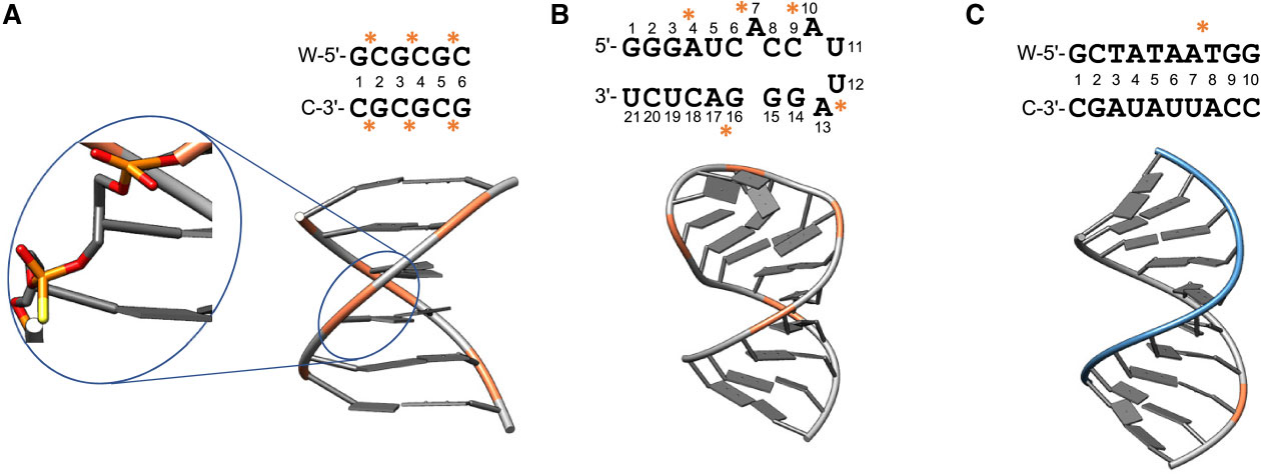
The studied structures of three representative PS-modified therapeutic nucleic acids. (**A**) PS analogues of B-DNA (PDB code 1D97), (**B**) PS modified RNA binding site for phage MS2 coat protein (PDB code 1D0T), (**C**) DNA:RNA hybrid duplex with a single PS moiety (PDB code 219D) shown in blue and grey. PS-ON sites are shown in orange ribbon. The location of the PS modifications is highlighted with ‘*’.

One of the most widely used modifications in oligonucleotide medicinal chemistry is the replacement of a phosphodiester oxygen in the backbone with a sulfur atom to produce phosphorothioate oligonucleotides (PS-ONs; Figure 1) (2). PS-ONs have similar overall chemical properties to the natural phosphodiester oligonucleotides (PO-ONs), retaining the anionic character of the backbone. The enhanced nuclease stability of the PS bond is accompanied by a lower binding affinity for complementary target RNA (3), and additional modifications in the sugar and/or base are commonly used to balance stability and affinity (4).

The development of therapeutic oligonucleotides typically requires an extensive, wet-lab trial-and-error process. Oligonucleotide sequence and chemistry are iteratively modified to optimize potency, usually measured in cell-based assays as the modulation of target transcript levels using quantitative reverse transcriptase-polymerase chain reaction (RT-PCR). Cellular potency results from a multitude of factors, including cellular uptake and intracellular trafficking of the ASO, basal target transcript turnover, ASO-transcript hybridization, and abundance and efficiency of RNaseH (5,6). Importantly, the hybridization process plays a critical role in determining not only selectivity towards the intended target sequence, but also the efficiency of degrading the target transcript (6). Computational techniques, such as molecular dynamics (MD) simulation, can provide mechanistic insights into the ASO–mRNA hybridization process at the atomic level, and thereby guide the optimization of ASO sequence and chemical modifications and facilitate a rational design of new PS-ONs with optimal on- and off-target binding characteristics.

Atomistic MD simulations require the definition of molecular mechanics (MM) force field parameters to quantify the potential energy of the simulated molecules over time (7). While force field parameters for protein, lipids and natural NAs have been extensively derived and validated, counterparts for chemically modified nucleic acid (NA) derivatives like PS-ONs are still under development (8,9). The first attempts to derive AMBER MM force field parameters for PS date back more than a decade and were usually limited to RESP fitting of charges (10), quantum-mechanical (QM) derivatization without explicit optimization to MM level of theory (11,12), or QM/MM optimization for specific interactions, e.g riboswitch and neomycin (13). As such, there is only limited information available from previous work regarding general PS AMBER force field parameters (14).

To facilitate atomistic simulations of therapeutic ASOs, we derived new force field parameters for PS-ONs and validated them against experimentally determined conformations. Three test cases were selected, representing both the DNA- and the RNA-based regions of the typical gapmer ASO design, as well the DNA-RNA binding: (i) a nucleic acid analogue of B-DNA (15), (ii) the RNA binding site for phage MS2 coat protein (16) and (iii) a model DNA/RNA hybrid duplex (17) (Figure 1). The three test cases were used to (a) assess the ability of our new parameters to reproduce experimental data; and (b) provide additional insights into the impact of PS modifications on nucleic acid conformational dynamics, by comparing simulations of PS-modified ONs with the corresponding phosphate-linked structures. We find that the new parameters can correctly describe the molecular geometries and interactions of the studied systems, thus supporting their application to further PS-modified oligonucleotide systems, and contributing to our understanding of the structural and functional consequences of DNA and RNA phosphorothioation. Our study provides insights into the effects of PS modifications on the conformational landscape and target binding propensity of ASOs. In particular, we observe that the impact of PS modification depends on the class of the nucleic acid molecule, with the most significant impact on the sequence specific conformational behavior of the example dsDNA system and more moderate effects in the studied ssRNA system. The presented force field parameters and our findings on the impact of PS modification on structural dynamics provide a foundation for further atomistic exploration of ON hybridization mechanisms, off-target and protein interactions, ultimately aiding the rational design of selective and potent antisense therapeutics.

## Materials and methods

### Parameterization strategy for phosphorothioate-modified therapeutic nucleic acids

Current state-of-the-art force fields for nucleic acids include parmBSC1 (18) for DNA-based and parmbsc0 + OL3chi (19) for RNA-based NAs. However, both force fields lack parameters for the PS function. We therefore derived the relevant force constant and equilibrium length parameters for the bond between the phosphorous and sulfur atoms, P–S, as well as the contributions from the two S–P–O angles involving two different oxygen atom types, and from the S–P–O–C dihedral angle (Supplementary Figure S1a).

To obtain initial topology and parameters files, the CGenFF Paramchem webserver (20,21) was interrogated using dimethyl phosphate (DMP; Supplementary Figure S1b). DMP was used as a template molecule since its atom types are consistent with those in nucleic acids (e.g. terminal carbon atoms are sp^3^ hybridized). The small dimensions of DMP allowed us to avoid bias from the steric influence of sugar groups on the molecule's potential energy surface during the energy scan of the torsional angle, thereby ensuring parameter transferability. Parameter and topology files for dimethyl phosphorothioate (DMPT), necessary for the successive parametrization stages, were then obtained from the corresponding DMP files by substituting the atom type O1P (DMP) with a new atom type named SPT (DMPT).

The initial DMPT conformation was energy-optimized with Gaussian09 at the HF/6–31G* level of theory before computing the electrostatic potential (ESP) partial charges (22) (using the keyword pop = mk iop(6/33 = 2.6/41 = 10.6/42 = 17)). These were then fitted into the restrained ESP (RESP) model (23) using the Antechamber program in AmberTools20. The DMPT topology file was updated with the RESP partial charges and used, alongside the parameters file and the QM-optimized geometry, to derive bond parameters through the force field toolkit plugin (24) of VMD (25) as follows: first, non-bonded van der Waals parameters were temporarily assigned to the atom type SPT by analogy from the type SG3O2 (i.e. thiolate sulfur (−1)). Then, the Hessian for vibrational mode analysis was computed with Gaussian09 (MP2/6–31G* level of theory,) before iteratively optimizing parameters for the S–P bond and the S–P–O angles 1 and 2 (using the default ffTK settings). Finally, the potential energy surface (PES) of the S–P–O–C rotatable bond was computed at the MP2/6–31G* level of theory with a pace of 15° between conformations, and the corresponding MM parameters were optimized iteratively to match the QM PES (Supplementary Figure S1c). The obtained topology and parameters files were used to build the DMPT psf file, which was then converted to an Amber prmtop file using ParmEd (26). Finally, the non-bonded Lennard-Jones parameters used in MD simulations were retrieved from the amber14sb force field.

### Preparation of phosphorothioate-modified therapeutic nucleic acids

To validate our derived force field parameters, we selected three different classes of nucleic acid molecules as model systems and identified suitable experimentally solved structures. The 3D-coordinates for PS-modified therapeutic NAs used in this study were retrieved from the Protein Data Bank (27): a PS analogue of B-DNA (PDB code 1D97 (15); (Figure 1A), the PS-modified RNA binding site for phage MS2 coat protein (PDB code: 1D0T (16); Figure 1B), and a partially phosphorothioated DNA/RNA hybrid duplex (PDB code: 219D (17); Figure 1C), respectively. The PS-ONs were prepared using Molecular Operating Environment (MOE) 2019.01 suite (28), including the addition of missing hydrogen atoms using the ‘Protonate3D’ tool (29). In more detail, the B-DNA system contained 3 chiral centers per strand, for a total of six chiral centers, in which the phosphate 5′ in each cytidine residue was replaced by *Rp*-PS. For the ssRNA system, *Rp*-PS modifications were located 5′ to every adenine residue. The DNA-RNA hybrid duplex contained a single chiral *Sp*-PS center in the DNA strand between A_7_ and T_8_. In force field development, chirality does not affect the results and the same parameters are assigned to (*R*) or (*S*) isomers.

To assess the impact of PS substitution on the conformational dynamics of nucleic acids, the corresponding natural diphosphate-bonded analog of each modelled system was prepared. For the RNA example, the experimentally determined structure was prepared using the same protocol as described for PS-modified NAs. For the two other systems, experimental structures of the PO-bonded analogs were not available; these were instead modelled by exchanging all sulfur atoms for oxygens.

### Molecular dynamics simulations

Classical molecular dynamics (MD) simulations were performed using the MD engine GROMACS v2022.1 (30) using parmBSC1 (18), parmbsc0 + OL3chi (19) and the combination of parmBSC1 (18) and parmbsc0 + OL3chi (19) force fields for the DNA, RNA, hybrid DNA–RNA, respectively.

The three test-case systems were solvated with TIP3P water (31) in cubic periodic boxes with a buffer distance of 15 Å to the walls. PS B-DNA was then neutralized by K^+^ counterions and additional K^+^/Cl^−^ counter ions were added to reach a physiological salt concentration of 0.15 M. PS RNA and PS DNA/RNA hybrid duplex were first neutralized by Mg^2+^ counterions and additional Na^+^/Cl^−^ counter ions added to give a physiological salt concentration of 0.10 M. Mg^2+^ has been used because it has been shown that Mg^2+^ ions play a significant role in RNA stability (32). Applying periodic boundary conditions, all systems were subjected to (a) energy minimization with 5000 steps of steepest descent, followed by (b) 500 ps equilibration with position restraints on heavy solute atoms (1000 kJ mol^–1^ Å^–2^) in the NVT and (c) 20 ns equilibration with position restraints on heavy solute atoms (1000 kJ mol^–1^ Å^–2^) in the NPT ensembles, adjusting temperature and pressure to 300 K and 1 atm. Releasing the restraints, 2 μs MD simulations were then carried out at constant pressure and temperature (1 atm and 300 K).

To evaluate the effect of PS substitution on the structural dynamics of the nucleic acid systems, we also performed MD simulations of the natural oligonucleotides (i.e. DNA, RNA and DNA–RNA hybrid duplexes without PS-modifications), following the described above protocols.

### Trajectory analysis

The generated MD trajectories were preprocessed using GROMACS v2022.1 (30) and CPPTRAJ program (33) in the AMBERTOOLS 16 software package. Cluster analysis was performed using the gromos method (34) on heavy atoms in the PS-and PO-ONs with a cutoff of 0.2 nm. Helical, groove and backbone parameters were derived using Curves+ and Canal (35) for each trajectory snapshot, collected every ps. The results are presented in several subsections, each focusing on a specific aspect of the structural dynamics of therapeutic nucleic acids with PS backbone modifications, i.e. (a) stability, (b) helical parameters and (c) torsional angle and sugar pucker analysis.

## Results

### Force field parameterization

AMBER is presently considered among the best-parameterized force fields for natural NAs. For this reason, we extended current AMBER natural-NA force fields to include PS parameterization. RESP partial charges for DMPT, the smallest entity able to describe the PS chemistry in the correct environment, were computed using a QM level of theory consistent with AMBER (*i.e*. Hartree-Fock, 6–31G*). We found reduced polarizability of the bonds between the central phosphorous atom and the adjacent heteroatoms part of the PS group compared to the natural PO backbone, in accordance with previous data (36). Lennard–Jones parameters for the sulfur atom of the PS were assigned by analogy from the thiolate atom type available in AMBER to maintain self-consistency in long-range interactions within the force field, although Lennard–Jones parameters from CHARMM36 have been previously suggested to better reproduce hydrogen bonds and solvent interactions than the corresponding AMBER non-bond parameters (13).

Considering the good transferability of CHARMM36 non-bond parameters, we decided to feed the QM-derived Hessian matrix to the optimization algorithm for CHARMM. In this way, we computed from scratch the equilibrium length, angle, and constants of the bond involving the PS sulfur atom (Supplementary Figure S1a). This thorough parameterization procedure was completed by a QM scan of the S–P–O–C dihedral angle and subsequent optimization to the MM level of theory. The MM potential energy surface of the torsional after the QM optimization remarkably overlapped with the QM energy profile, indicating a substantial improvement of the initial dihedral parameters (Supplementary Figure S1b).

### Model system preparation and stability analysis

We retrospectively evaluated the applicability domain and accuracy of our PS-modified force field parameters by assessing how well they reproduce experimentally solved structures. Specifically, as described in the following sections, we selected for analysis three representative model systems for different classes of nucleic acids: a B-DNA analogue (15), a DNA:RNA hybrid duplex (17) and a single-stranded RNA (16) containing a single-nucleotide bulge and a four-nucleotide hairpin (Figure 1). We compared the conformational dynamics of the PS-modified and the corresponding natural, fully PO-bonded systems, addressing important questions within the common chemical modifications used in therapeutic antisense oligonucleotides. The B-DNA analogue contains PS modifications on all cytosines (six in total), the RNA hairpin analogue contains the modifications on all adenines (five in total), and the DNA:RNA duplex contains the modification on the thymine nucleotide of the last TA base pair (Figure 1).

To assess the structural stability of the PS analogues, we first calculated the root-mean-square deviation (RMSD) (Supplementary Figures S2-4) of the phosphorous backbone atoms throughout the MD simulation, with respect to the initial minimized structure. The low and relatively constant RMSD values (1.25 ± 0.30, 2.41 ± 0.43 and 2.33 ± 0.41 Å for the B-DNA, DNA:RNA duplex and ssRNA respectively) indicate overall structural stability during the 2 μs MD trajectory (Video S1, S2 and S3). For comparison, the same analysis was performed for the corresponding natural ONs, showing similar RMSD values and low fluctuations (1.17 ± 0.32, 2.36 ± 0.42, 2.36 ± 0.42 Å for the B-DNA, DNA:RNA duplex and ssRNA, respectively). The NMR solved conformation ensembles for PS-modified and natural ssRNA contained ten and twenty-three structures, respectively. We used the first structure of each ensemble as starting points for the MD simulations. The fluctuations observed in the MD trajectories follow from conformational changes in the loop region, in accordance with the NMR-derived ensembles (Video S3).

**Figure 2.**
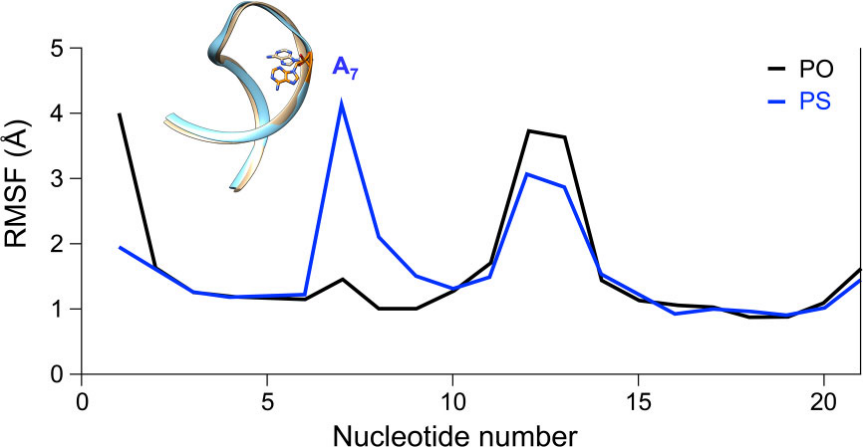
RMSF of residues along the PS-modified (PDB code: 1D0T) and natural ONs (PDB code: 1D0U), in blue and black respectively. The differently oriented A_7_ base, as observed in the most populated cluster during 2 μs classical MD simulation for the PS, in orange, and natural, in tan, systems.

Next, we analyzed root mean square fluctuation (RMSF) to address which regions of the oligonucleotides exhibit the largest fluctuations and to see if any of the fluctuations were altered by PS modification. For the DNA and DNA:RNA hybrid systems, the impact of PS modifications was negligible (Supplementary Figures S5 and S6). For ssRNA, a key difference between the PS-substituted (PDB code: 1D0T) and natural (PDB code 1D0U) structure in the MD simulations is the conformation of the bulged A_7_ nucleotide (Figure 2). In the natural ssRNA (Video S4) the A_7_ nucleotide intercalates between its flanking C_6_–G_16_ and C_8_–G_15_ base pairs, stacking between the pyrimidine of C_6_ and C_8_. In the PS modified system, the A_7_ experiences larger fluctuations, and fraying where the stacking is lost (Video S5). For the most populated cluster of conformations, the A_7_ nucleotide is located in the major groove, with the flanking base pairs stacking each other (i.e. C6–G16 and C8–G15 base pairs stacking).

To further validate our derived PS-modified force field parameters, the inter-strand phosphate distances for each base pair were calculated for both the PS-modified and natural-ONs systems. The distances were similar and stable and in accordance with those in the experimental structure (Supplementary Figures S7-9). For the ssRNA systems, the inter-strand phosphate distances showed larger fluctuations within the loop region than in the stem region, particularly in position 11–13, as evidenced by a greater standard deviation (Supplementary Figure S9). Also, in the PS system, the U_11_–U_12_ base stacking is disrupted by the formation of independent A_10_–U_11_ and U_12_–A_13_ base stacking events (Supplementary Video S6). It is worth noticing that the same structural rearrangements occur also for the natural ONs (Video S7). In both systems (a) A_10_ stacks on the C_9_–G_14_ base pair, (b) U_11_ stacks on A_10_ and (c) U_12_ stacks on A_13_, moving away from the loop.

### Helical parameters

The conformational dynamics of nucleic acids have profound effects on their function and interactions with other macromolecules. Rearrangements in nucleic acid conformation can be characterized by several helical parameters that describe the relative movement of the nucleotides. Thus, to address in-depth if PS modification impacts conformational sampling in the studied nucleic acid systems, we next analyze such inter-base pair helical parameters.

**Figure 3.**
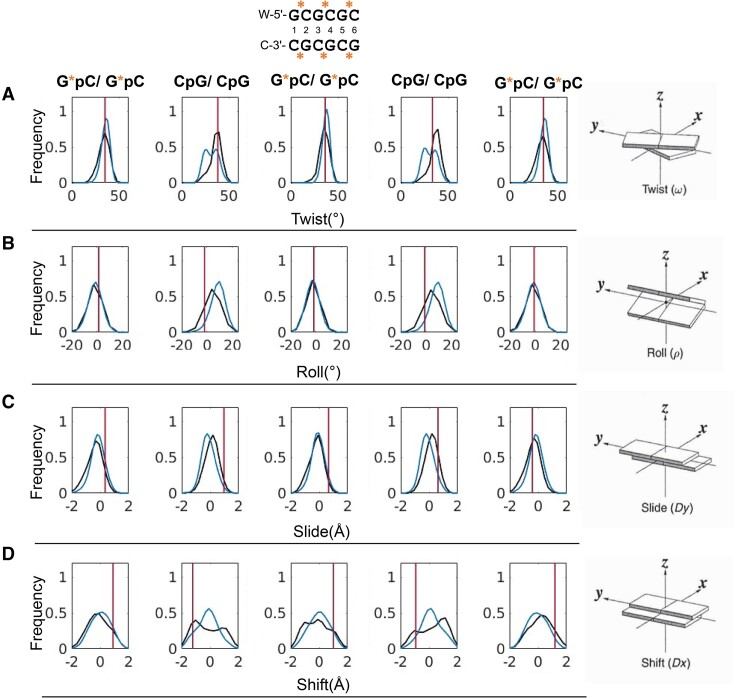
Distributions of (**A**) twist, (**B**) roll, (**C**) slide and (**D**) shift parameters for PS-modified (blue) and natural ONs (black), respectively. Red lines correspond to the values for the experimental structure (PDB code: 1D97).

For the B-DNA systems (Figure 3), we observed differences in the parameters *twist*, *roll*, *slide* and *shift* resulting from the PS modification. Interestingly, in contrast to natural B-DNA, the PS substitution resulted in a bimodal twist distribution for the corresponding CpG base pair steps, indicating that they can shift between a high and low twist state. The CpG step in the purine..pyrimidine (R..Y) tetranucleotide environment in natural B-DNA is significantly more rigid (37). Analysis of the experimental structure shows one of the CpG steps in a higher twist state, whereas the other is in a lower twist state. Thus, the bimodality captured in our simulations is reasonable. Changes in CpG twist behavior lead to an increase in the roll angle, a slight decrease in the slide and a unimodal shift distribution. In turn, the GpC steps become more rigid, which is shown by narrower distributions (Figure 3).

It has been suggested through QM calculations (38) that PS modifications destabilize the B-form of DNA due to steric effects caused by the larger S-atom. To assess a potential duplex-destabilizing effect of the PS modifications, we also analyzed the helical parameters, helical rise and helical twist (*h-rise*, *h-twist*), *x*-displacement (*xdisp*) and incline (*incl*) (Supplementary Figures S10-11). *h-rise* and *h-twist* differ from *rise* and *twist* in that they describe the translation and rotation of two adjacent base pairs in relation to the helical axis instead of relative to a base pair step (39). For B-DNA structures these parameters will provide similar values (Supplementary Figure S11). In contrast, when the base pairs become more inclined and DNA approaches an A-form conformation, *h-rise–rise* and *h-twist–twist* will differ significantly. Supporting that DNA with PS-modifications adopt the B-form, *h-rise* and *h-twist* mirror *rise* and *twist* (Supplementary Figure S11). However, we observe a slight decrease in *xdisp* and a slight increase in inclination for all base pairs in PS-modified DNA, indicating some steric influence from the sulfur atom. The decrease in the twist of the CpG steps also points towards such a steric effect, although rather insignificant.

**Figure 4.**
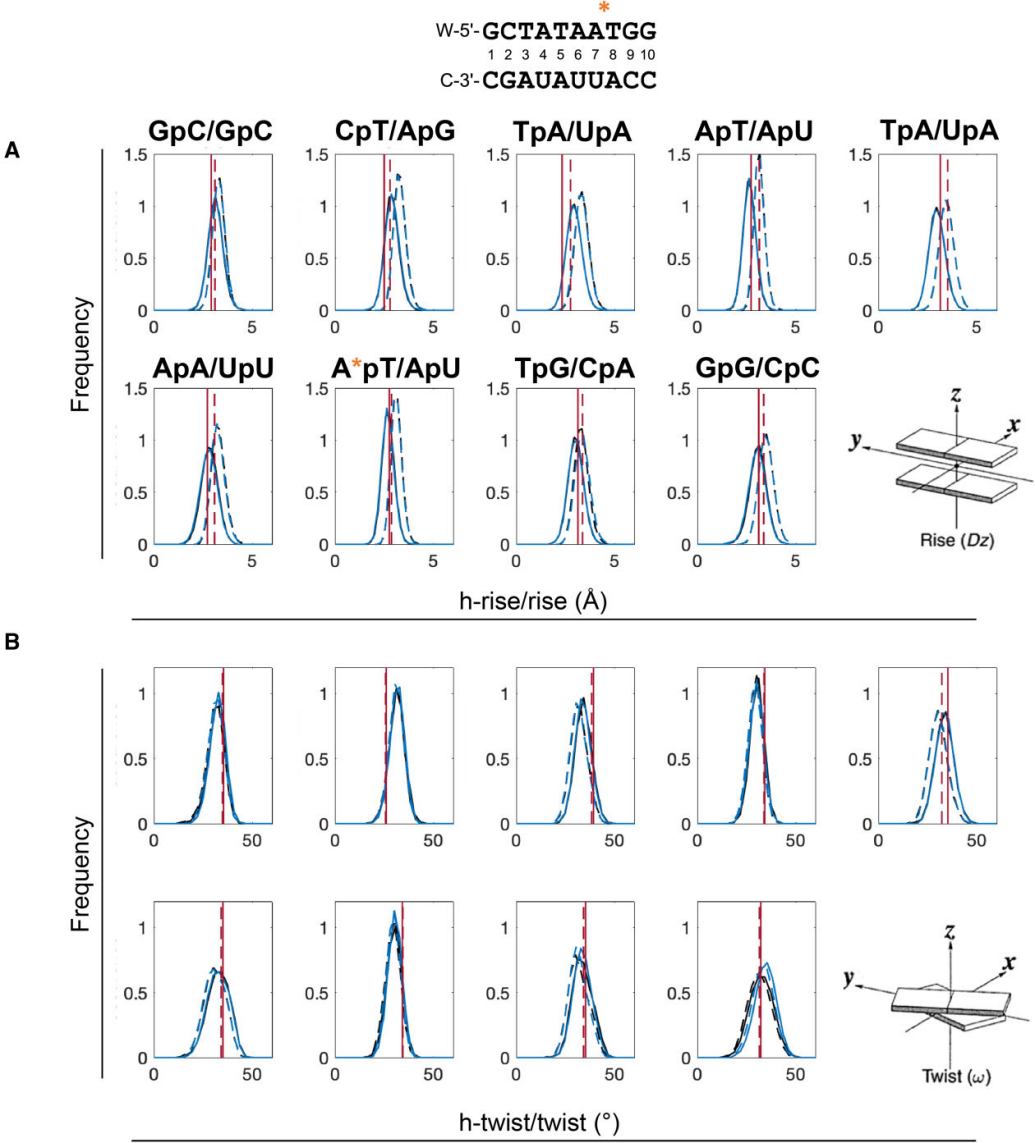
Comparison of (**A**) h-rise (bold lines) and rise (dashed lines) and (**B**) h-twist (bold lines) and twist (dashed lines). Large deviation is common for the A-form conformation. PS-modified (blue) and natural ONs (black), respectively. Red line illustrates the value for the NMR structure 219D. The location of the PS modifications is highlighted with ‘*’.

For the DNA:RNA hybrid with a single PS substitution, the helical parameters (Supplementary Figure S12) were similar for the PS and PO system, which is also consistent with the experimental data (shown with a red line for each base pair step). This suggests a dependence of conformational dynamics on the number of PS modifications introduced, and that multiple PS substitutions are needed for substantial impact. Under physiological conditions, DNA is predominantly in the B-form whereas RNA is in an A-form. We again analyzed helical parameters to explore more thoroughly which conformation the DNA:RNA hybrid duplex attains (Figure 4 and Supplementary Figure S13), and observed values for *h-rise* (<3.0 Å) lower than for *rise* (>3.0Å), *h-twist* 1–2° greater than *twist*, and *xdisp* and *incl* >−3.0Å >10°, respectively. Collectively, these results indicate that the DNA:RNA hybrid adopts an A-form conformation (40).

**Figure 5.**
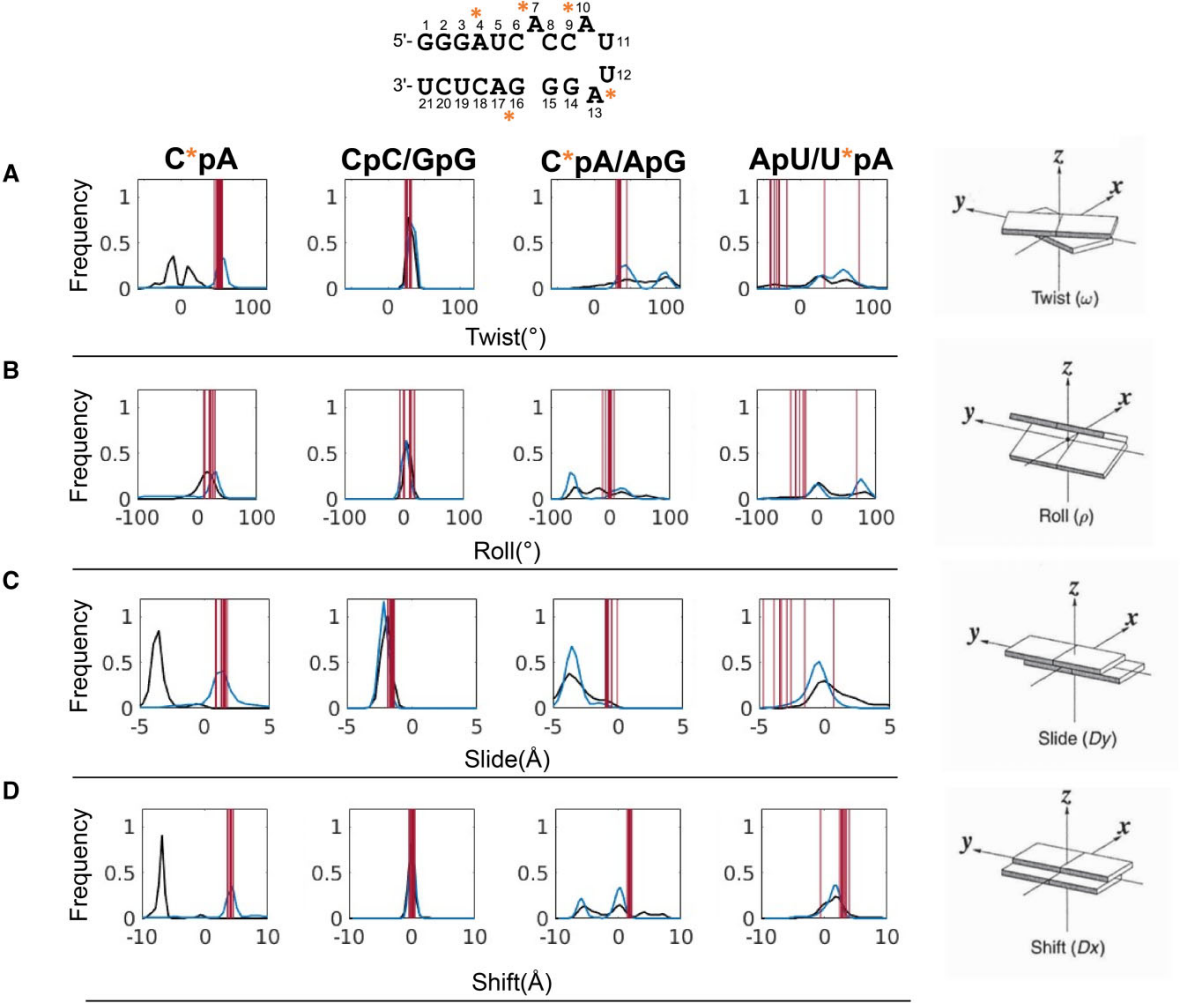
Distributions of (**A**) twist, (**B**) roll, (**C**) slide and (**D**) shift for PS-modified (blue) and natural ONs (black), respectively for the base pair steps highlighted with bold text. Red lines represent experimental helical parameters for each base pair step for the NMR structure 1D0T.

For the ssRNA system, we observed differences in twist, slide and shift, between the PS-modified and natural systems, in proximity to the bulged A_7_ nucleotide (Figure 5 and Supplementary Figure S14) and for the bases within the 4-bases hairpin loop. However, it should be noted that the bases within the loop do not form actual base pairs, therefore the differences could be due to the general high flexibility of loop bases rather than an impact of PS modifications.

To characterize the preference for A- or B-form conformations, we also analyzed the *h-rise*, *h-twist*, *incl* and *xdisp* parameter distributions (Supplementary Figures S15 and S16). *h-rise* was lower than *rise* (<3.0 Å) whereas the *h-twist* and *twist* distributions were approximately equal. This suggests that the hairpin adopts a conformation more resembling the A-form. Interestingly, for the PS-modified system *h-rise* is higher, and the distributions are narrower for the base pair steps near the bulge and within the hairpin; this suggests that the PS-modified RNA molecule has a more specific and restricted conformation. The *xdisp* and *incl* distributions suggest an A-form for the molecules (40), with values <−3.0 Å and >15° respectively. However, near the bulge and for the hairpin residues the distributions become broader which capture both the A-form and B-form conformation.

### Torsional angles and sugar puckers

The PS modifications are located at the nucleic acid backbone and are thought to play important roles in interactions between PS-ONs and cellular and plasma proteins. Changes to the structural dynamics of ONs induced by PS modifications could be part of the explanation for altered binding properties. Thus, as a final analysis of the potential impact of the PS modifications on the conformational dynamics, we also look at how the backbone dynamics is altered by phosphorothioation. Specifically, we analyzed the backbone torsional angles ϵ and ζ, χ, α/γ and sugar pucker distributions. The ϵ and ζ torsions are particularly interesting for the B-form, as transitions in these angles provide the BI (ϵ and ζ < 0°) and BII (ϵ and ζ > 0°) conformations.

**Figure 6.**
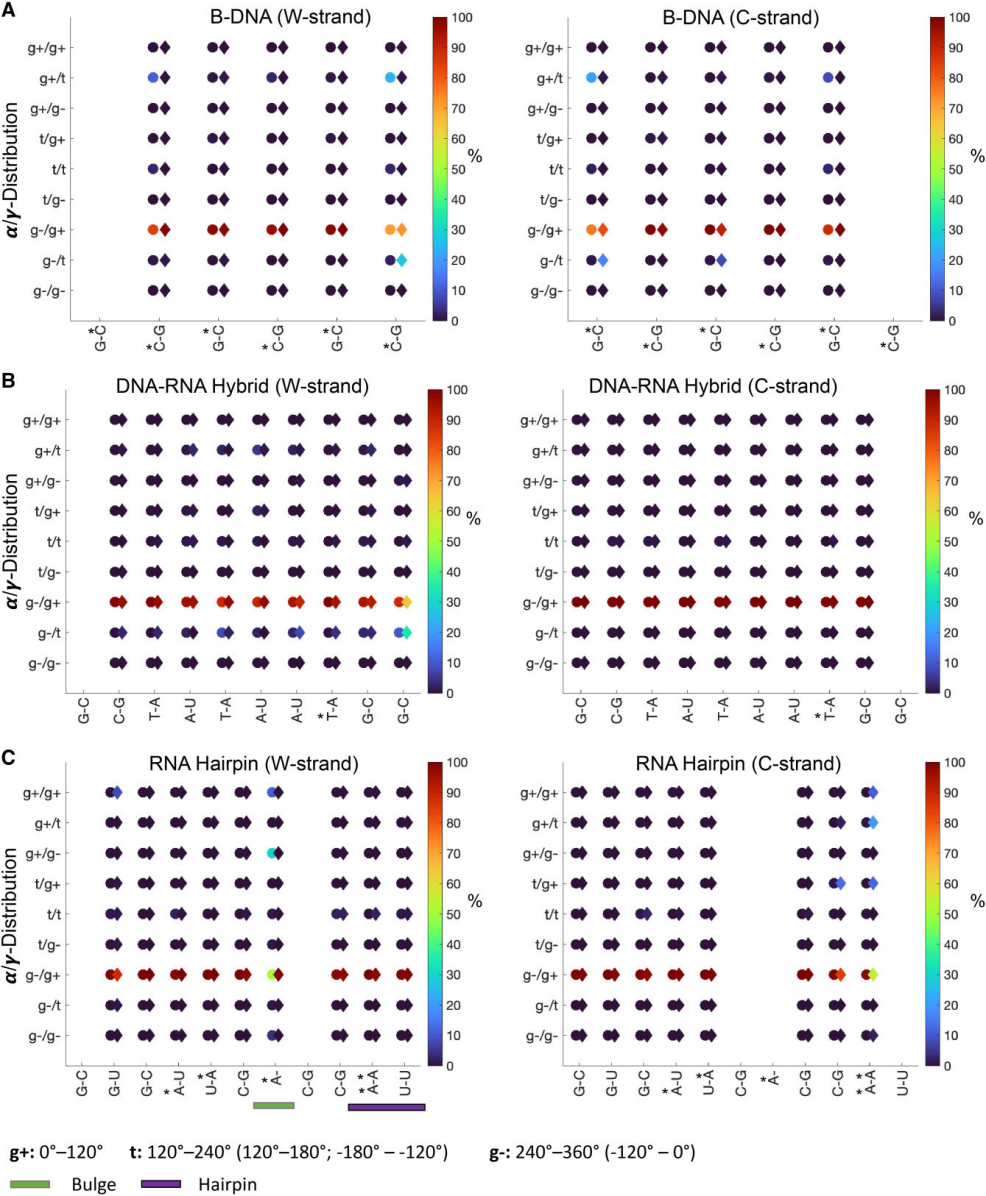
α/γ-distributions (%) for (**A**). B-DNA (**B**). DNA:RNA hybrid duplex (**C**). Hairpin–RNA. PS-modified systems are denoted with colored circles and natural systems are denoted with colored diamonds. The location of the PS modifications is highlighted with ‘*’.

**Figure 7.**
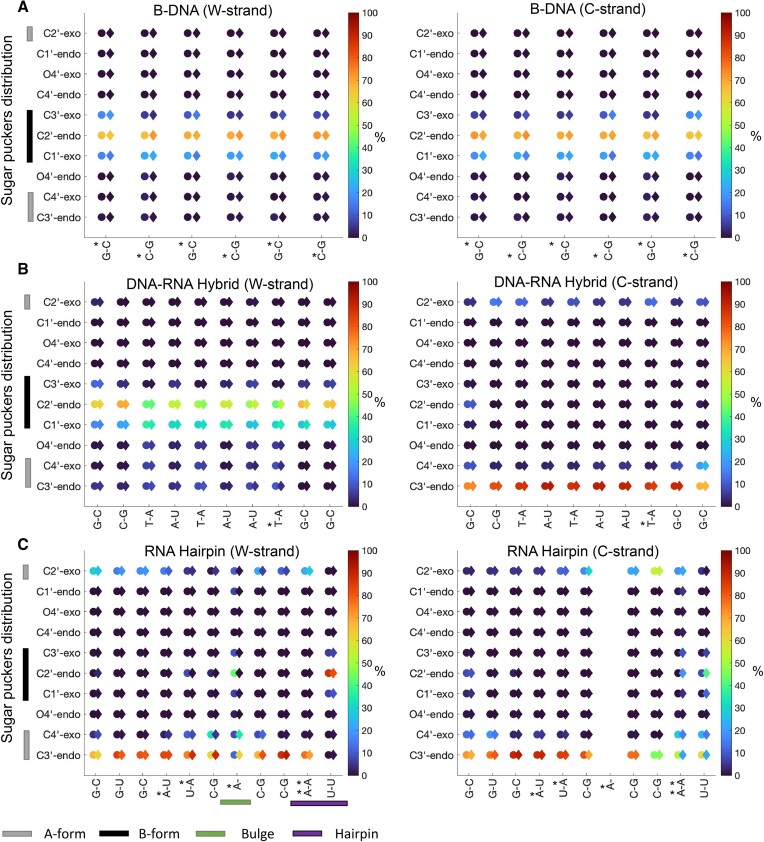
Sugar pucker distributions (%) for (**A**). B-DNA (**B**). DNA:RNA hybrid duplex (**C**). Hairpin–RNA. PS-modified systems are denoted with colored circles and natural systems are denoted with colored diamonds. The location of the PS modifications is highlighted with ‘*’.

For the B-DNA systems, we find that the PS modifications impact the ϵ and ζ distributions (Supplementary Figures S17-18) with, for instance, higher populations of ζ > 0°, which explains the differences observed in the helical parameters. Nevertheless, all base pairs for both systems are predominantly in the BI conformation. No major alterations resulting from the presence of PS modifications were seen in the chi-angle (Supplementary Figure S19), the α/γ, and the sugar pucker distributions (Figures 6A-7A and Supplementary Figures S26A and S27A): the χ-angle distributions became slightly narrower, and the α/γ distributions show that the base pairs are in the canonical g–/g+ state; only small differences are observed for the outer base pairs which could be due to that these base pairs in long simulations are more susceptible to base pair fraying. The sugar pucker distributions show that the base pairs spend most of the time in the C2′-endo conformation (∼70%), but also transition to the C1′-endo and C3′-exo conformations; all characteristic for the B-form (41).

In contrast, we observed no significant impact of the PS modification for the DNA:RNA systems. The α/γ distributions show that both strands are in the g–/g+ conformation (Figure 6B and Supplementary Figure S26B). Notably, the ϵ, ζ and χ-angles and the sugar puckers show that DNA and RNA keep their intrinsic characteristic structures also when forming the hybrid duplex (Supplementary Figures S20–S22). The DNA strand (w-strand) exhibits broader ϵ and ζ distributions with some transitions to ϵ, ζ > 0. The χ-angle distributions for the DNA strand are also broader, with the mean around –120°, whereas the χ-angle distributions for the RNA strand (c-strand) are narrower and more negative at ∼–150°. The sugar puckers (Figure 7B and Supplementary Figure S27B) for the DNA strand also show that the nucleotides are predominantly in the C2′-endo conformation whereas for the RNA strand, they are in the C3′-endo conformation. Nonetheless, the nucleotides of the DNA strand show a stronger preference for the C1′-exo conformation compared to the B-DNA structure (PDB ID: 1D97). This agrees with previous computational analyses of DNA:RNA hybrids (42).

In the ssRNA systems, differences in helical parameters were observed near and within the bulge and within the hairpin. In line with these observations, analysis of the torsions showed transitions ϵ, ζ > 0 (Supplementary Figures S23-S24). The α/γ distributions indicate that most of the nucleotides in the ssRNA systems adopt the g–/g+ conformation (Figure 6C and Supplementary Figure S26C). Transitions to the g+/g– and g+/g+ conformations were seen for the A7 bulge in the PS-modified system and for the hairpin residues in the unmodified system. The sugar pucker distributions (Figure 7C and Supplementary Figure S27C) show that most nucleotides are in the C3′-endo conformation characteristic of the A-form. However, in the PS-modified system, the A7 nucleotide appears to prefer the B-form characteristic C2′-endo conformation. The B-form C2′-endo conformation also contributes to a less negative χ-angle, ∼–114°, for the A7 base of the PS system compared to the unmodified system ∼−170° (Supplementary Figure S25).

## Discussion

We have developed from scratch MD force field parameters for the extensively used PS moiety, enabling accurate all-atom simulations of PS-modified therapeutic nucleic acids and contributing to our understanding of the structural and functional consequences of phosphorothioation. In validating the force field parameterization against relevant experimentally determined structures representing PS-modified RNA, DNA and hybrid RNA:DNA duplexes, our results shed light on how PS substitution affects which conformations a modified oligonucleotide preferentially adopts, in turn influencing which interactions are possible and/or likely with target- and off-target biomolecules including mRNA and cellular proteins (43,44). Importantly, conformational sampling preferences affect both the target engagement process by defining if extensive, and potentially energetically non-favored conformational rearrangements are needed for duplex formation, and the likelihood for release from intended, and unintended–partially mismatching–sequences in the target transcriptome (6). By providing access to a well-validated set of force field parameters compatible with current state-of-the-art nucleic acid force fields (18,19), we open for further exploration into the atom-level events that govern binding thermodynamics and –kinetics of therapeutic oligonucleotides, and their selectivity towards their intended target sequences.

The new insights gained into the conformational dynamics of NAs align with, and extend, static-structure derived observations in the studies from which the experimental structure data were obtained (11–13). While we find that a single PS substitution had marginal effects on a DNA:RNA hybrid duplex structure, substantial conformational changes resulted from the more extensive PS substitution in the studied PS RNA and PS B-DNA systems. The B-DNA system exhibited differences in the inter-base pair helical parameters *twist*, *roll*, *slide* and *shift* between the PS-modified and natural NA. Specifically, we found that PS modification resulted in a bimodal *twist* distribution for the CpG base pair steps in the studied structure, indicating that they can alternate between high- and low-*twist* states. This observation has implications for DNA-protein interactions and for duplex formation between therapeutic, DNA-based ASOs and their nucleic acid targets. For example, such sampling of multiple discrete conformational states could contribute to the reduced affinity for RNA targets that results from PS substitution (3,45). Notably, discrete twist states are also observed in the two CpG steps in the corresponding experimental structure, providing experimental support for the bimodality captured in our simulations.

For the model PS-modified ssRNA system, we observed larger fluctuations in its bulged A_7_ nucleotide compared to in the corresponding natural, phosphodiester-based oligonucleotide. In the latter, A_7_ intercalates between its flanking C_6_–G_16_ and C_8_–G_15_ base pairs, stacking between the pyrimidine of C_6_ and C_8_. However, in the PS-modified system A_7_ exhibits larger fluctuations, in which stacking is lost. This suggests that PS modifications can affect RNA conformational dynamics and interaction potential. Analysis of helical parameters indicated effects from phosphorothioation that were greatest near and within the bulge and hairpin regions in the modelled ssRNA system. There, differences were seen in *twist*, *slide* and *shift* between the PS-modified and natural systems. Notably, *h-rise* was higher in the PS-modified system, and for the base pair steps near the bulge and within the hairpin the *h-rise* distributions were narrower, suggesting that the PS-modified RNA molecule has a more specific and restricted conformational sampling, again relevant for the conformational dynamics and hybridization potential for RNA-containing therapeutics.

In parallel with our effort, an alternative set of amber-compatible force field parameters for the PS backbone modification was recently presented by Genna *et al.* (46), derived using a different parameterization strategy. Comparing our force field parameters (partial charges, bond lengths, bond angles and dihedral parameters) with those presented in Genna *et al.*, analogies and differences are seen. Atom partial charges were generally consistent between the two parameter sets; Genna *et al.* assigned slightly less positive partial charge to the P atom (+0.6258e versus +0.9300e), but with the same trend in the PS and the neighboring C3′ and C5′ carbons as in our parameter set (Supplementary Table S1). Due to its central position in the PS group the phosphorous does not form direct hydrogen bonds and, hence, this difference is unlikely to significantly affect simulation outcomes. All other partial charge assignments were similar (absolute differences ranging 0.0627–0.1309e). Likewise, P–S bond parameters were very similar (equilibrium length *r*_eq_ differing by only 0.018 Å, i.e. less than 1/50 of Å) (Supplementary Table S2). Both parameterization approaches accounted for a greater bond length P–S than the corresponding P–O bond in the natural phosphate analog.

Due to differences in the parameterization approach, parameters for the angles involving the newly introduced sulfur atom (*i.e*. type S2) were more substantially different (Supplementary Table S3). We performed from-scratch parameterization of the bonds and angles involving the sulfur, assigning equilibrium angles of 121.171° and 110.965° respectively to the two P-centered angles, while Genna *et al.* instead assigned these parameters by analogy from the phosphate angle O2–P–OS (i.e. the angle formed by the sp^2^ oxygen, P, and the sp^3^ oxygen atoms; with a value of 108.230° for both angles) (44); thus, their parameters define a symmetry that is not found in other AMBER force fields (*e.g*. the nucleic acid phosphate group has equilibrium angles of 119.9° and 108.230°).

Different approaches were also taken in the optimization of dihedral parameters. Our reference structure, DMPT, is the smallest possible fragment describing the exact atom types involved in the PS modification; *de facto*, we considered the O3′ and O5′ oxygens as the same atom type in accordance with their hybridization (i.e. sp^3^) and that they are both covalently bound to sp^3^ hybridized carbons (C3′ and C5′). Thus, we only optimized a single dihedral, namely that involving the new sulfur atom type, while Genna *et al.* (44) reoptimized two dihedral angles that did not comprise the sulfur, and which are already included in parm99. The use of different reference structures for the optimization precludes strict side-by-side comparisons of the dihedral parameterization results.

The fact that substantial similarities are seen in the two force field parameter sets, despite having been derived through different parameterization strategies, strengthen the reliability of both simulation protocols. It is still possible that minor differences may translate to divergent simulation outcomes, and as for any force field parameterization, validation across multiple data sets and applications in future studies will be needed to confirm the optimal parameters. Importantly however, while addressing different research questions, our two studies also confirm specific observations regarding the conformational preferences of PS-modified oligonucleotides. For example, mutually corroborating results were obtained regarding the conformational preference of DNA:RNA hybrid duplexes substituted with *Sp*-chirality PS: in accordance with our results (Figure 4 and Supplementary Figure S13), Genna *et al.* report an A-like conformation for *Sp-*PS, while *Rp* chirality resulted in an A/B intermediate conformation and a ensuing higher thermal stability, (46) also aligning with previous experimental studies in supporting an order of thermal stability PO > *Rp*-PS > *Sp*-PS (47–51).

Additional PS parameterization philosophies are summarized in Supplementary Table S4 and compared to our strategy and that of Genna *et al.* (46) A variety of approaches have been employed, including different model structures (DMPT (13,52) or dinucleotides (46), and optimizing only specific aspects of the PS functionality (for example, only fitting atomic partial charges using the RESP approach, or only optimizing bond angles), while assigning other parameters by proxy from generalized force field settings (Supplementary Table S4). Notably, the derived parameter values are typically not presented on a level that allows comparison to other approaches, or application to new systems. Where available, we present parameter values and compare them to those developed here (Supplementary Tables S1-S3). To facilitate future simulation studies of PS-modified oligonucleotide systems, and further force field development, our complete parameters and step-by-step implementation instruction are available online (see Data availability below).

In conclusion, our analyses demonstrate the capacity of the derived force field parameters to accurately simulate conformational dynamics across a range of NA systems, including DNA, RNA and mixed duplexes, and indicate a significant impact of phosphorothioation on conformational sampling in DNA- as well as RNA-based structures. In the context of therapeutic antisense oligonucleotides that typically contain regions mimicking both these chemistries, our results provide additional rationale for the observed reduction in duplex stability that typically results from phosphorothioation (42,43). Given the demonstrated importance of well-balanced ASO–mRNA affinity for achieving optimal potency and therapeutic efficacy, (6) and the massive experimental efforts needed to optimize these and other pharmaceutical properties, the ability to accurately simulate the molecular processes determining affinity and selectivity will be an important component in a rational, mechanism-driven development of new backbone modifications for improved oligonucleotide therapeutics.

## Supplementary Material

lqae058_Supplemental_Files

## Data Availability

Description of the parameterization strategy for PS-modified therapeutic nucleic acids, and files related to the system preparation, MD trajectories and analysis, are provided freely accessible at https://doi.org/10.5281/zenodo.7872488.
