# Supplementary material for: Structural dynamics of therapeutic nucleic acids with phosphorothioate backbone modifications
Source: NAR Genom Bioinform. 2024 May 25;6(2):lqae058. doi: 10.1093/nargab/lqae058 (PMC11127634; doi:10.1093/nargab/lqae058)
Supplement: lqae058_Supplemental_Files [file lqae058_supplemental_files.zip › Carlesso_Structural dynamics of PS oligonucleotides_SUPPLEMENTAL MATERIAL_12_01_24.pdf]

Supplementary Material

Structural dynamics of therapeutic nucleic acids with phosphorothioate backbone modifications

Antonio Carlesso[1], Johanna Hörberg[2], Giuseppe Deganutti[3], Anna Reymer[2] , and Pär Matsson[1,4, *]

[1]Department of Pharmacology, Sahlgrenska Academy, University of Gothenburg, Box 431, SE-405 30 Gothenburg, Sweden

[2]Department of Chemistry and Molecular Biology, University of Gothenburg, Box 462, SE-405 30 Gothenburg, Sweden

[3]Centre for Health and Life Sciences, Faculty of Health and Life Sciences, Coventry University, Coventry, UK.

[4]SciLifeLab, University of Gothenburg, Sweden

[*]To whom correspondence should be addressed. Phone: +46-31-786 4932. Email: par.matsson@gu.se

Table of Contents

**Figure S1**	S3
**Figure S2**	S4
**Figure S3**	S5
**Figure S4**	S6
**Figure S5**	S7
**Figure S6**	S8
**Figure S7**	S9
**Figure S8**	S10
**Figure S9**	S11
**Figure S10**	S12
**Figure S11**	S13
**Figure S12**	S14
**Figure S13**	S15
**Figure S14**	S16
**Figure S15**	S17
**Figure S16**	S18
**Figure S17**	S18
**Figure S18**	S19
**Figure S19**	S19
**Figure S20**	S20
**Figure S21**	S21
**Figure S22**	S22
**Figure S23**	S23
**Figure S24**	S24
**Figure S25**	S25
**Figure S26**	S26
**Figure S27**	S27
**Table S1**	S28
**Table S2**	S29
**Table S3**	S30
**Table S4**	S31
**References**	S32

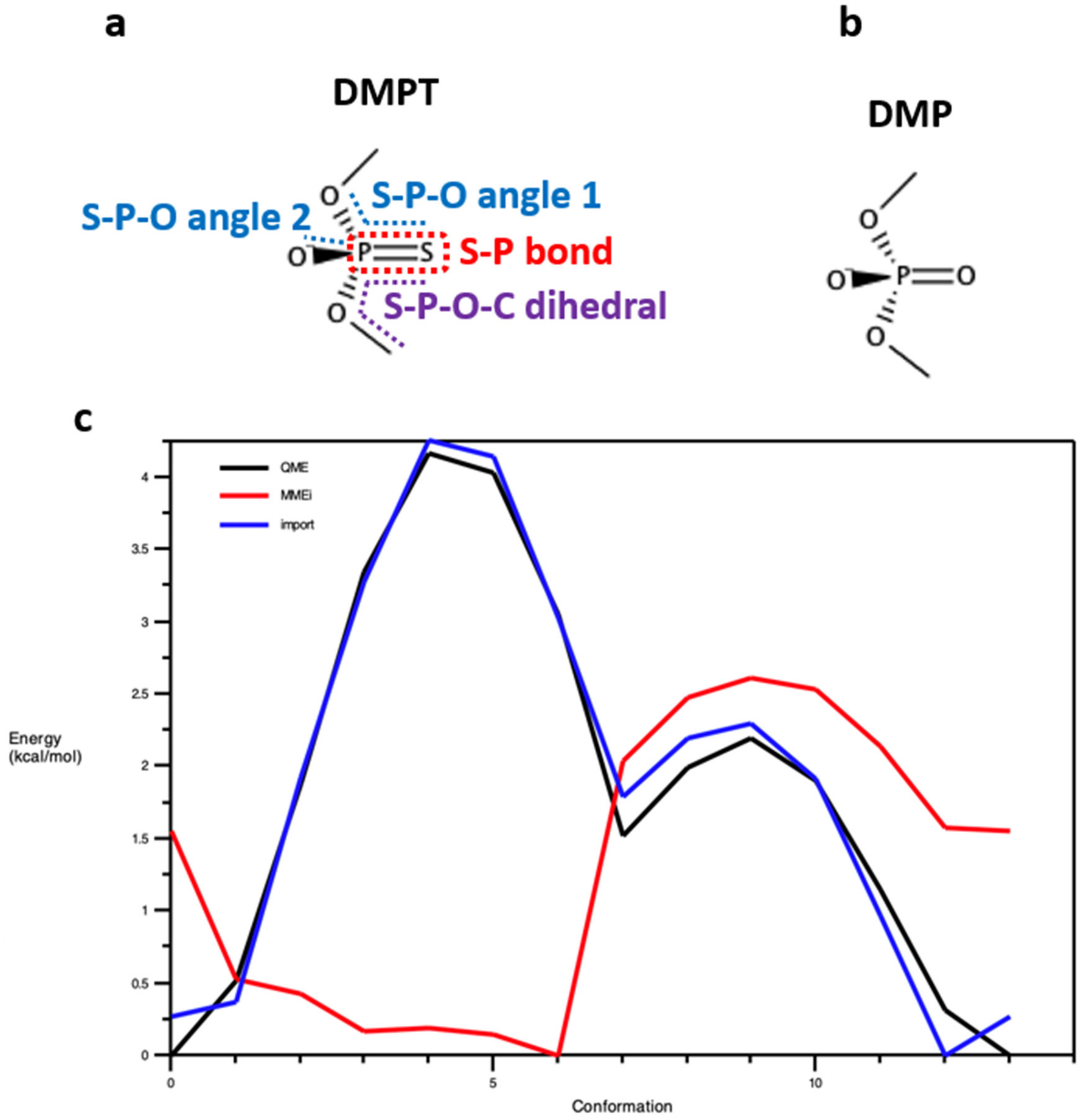

Figure S1. Parameterization of DMPT. a) The molecular mechanics parameters for the bond (S-P bond), angles (S-P-O angles 1 and 2), and dihedral (S-P-O-C dihedral) involving the sulfur atom were derived from the template molecule DMP (**b**); **c**) the PES of the optimized S-P-O-C dihedral (blue line) overlaps with the QM-derived PES (black line) and notably diverges from the original parameters of the template molecules DMP (red line).

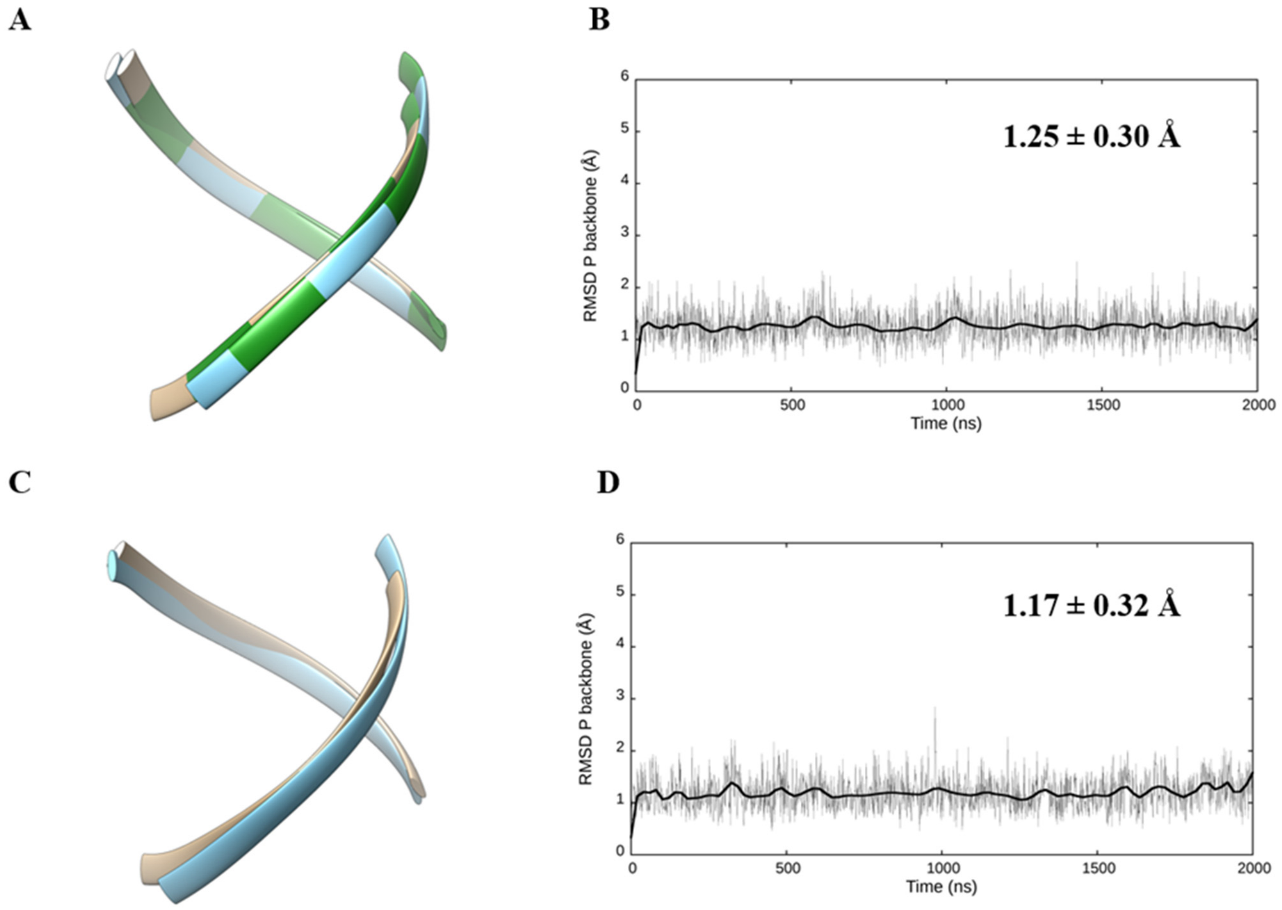

Figure S2. Structural data from the MD simulations. (A) Superposition of the representative structure of the most populated cluster during 2000 ns MD in blue, onto the crystallographic structure (PDB ID: 1D97) of the phosphorothioate analogue of B-DNA in tan. (B) Evolution of RMSD of the phosphorous backbone atoms during the 2000 ns MD simulation. The nucleotides with the phosphorothioate-modified backbone are shown in green. (C) Superposition of the representative structure of the most populated cluster, in blue, onto the initial structure of natural B-DNA in tan. (D) Evolution of RMSD of phosphorous backbone atoms during the 2000 ns classical MD.

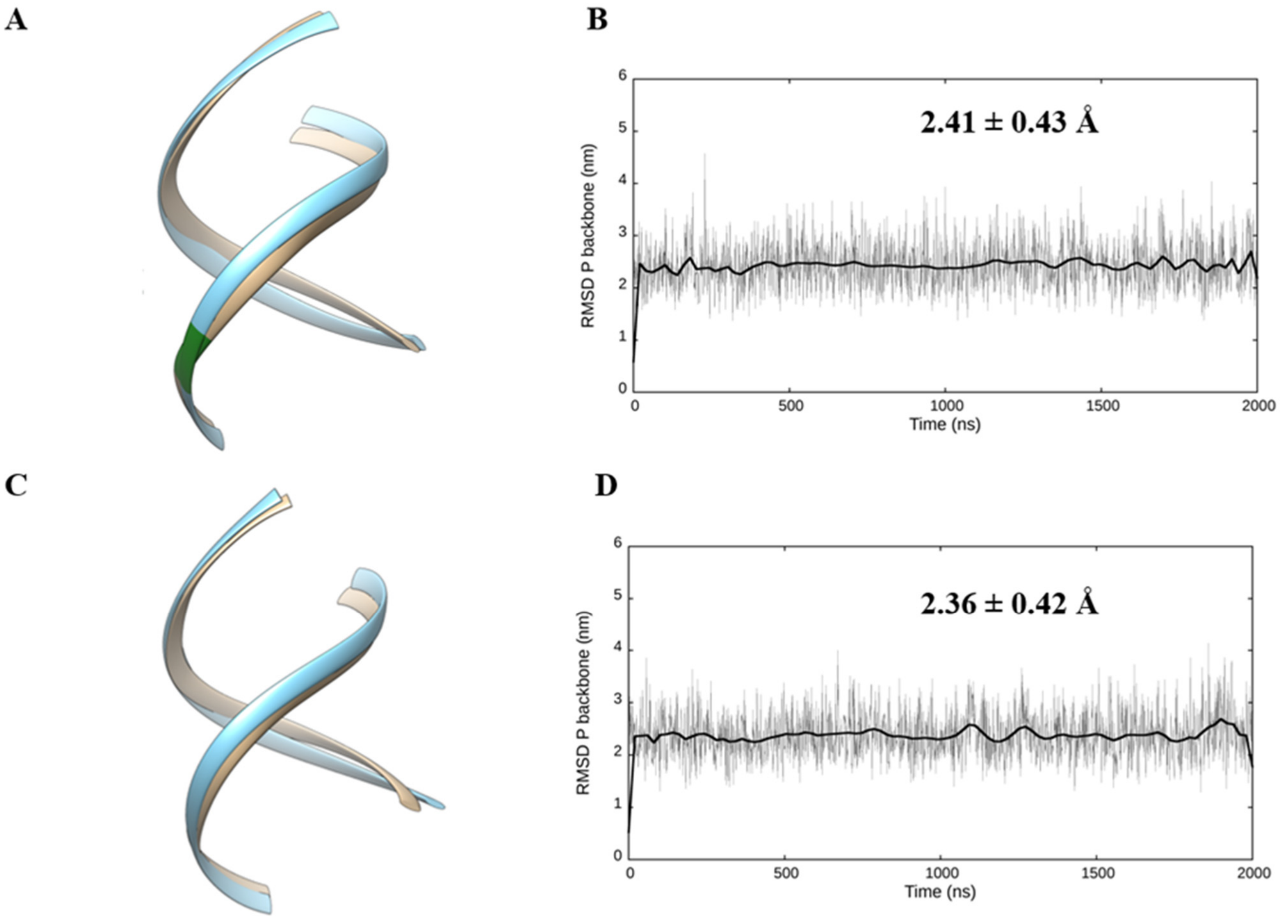

Figure S3: Structural data from the MD simulations. (A) Superposition of the most populated cluster during 2000 ns Classical MD (blue), onto the crystallographic structures (tan) of Phosphorothioate analogues of mixed DNA-RNA. (B) RMSDs of phosphorous backbone atoms during the 2000 ns classical MD. Position of the PS is shown in green in (A). (C) Superposition of the most populated cluster onto the initial structures (tan) of natural mixed DNA-RNA. (D) RMSDs of phosphorous backbone atoms during the 2000 ns classical MD.

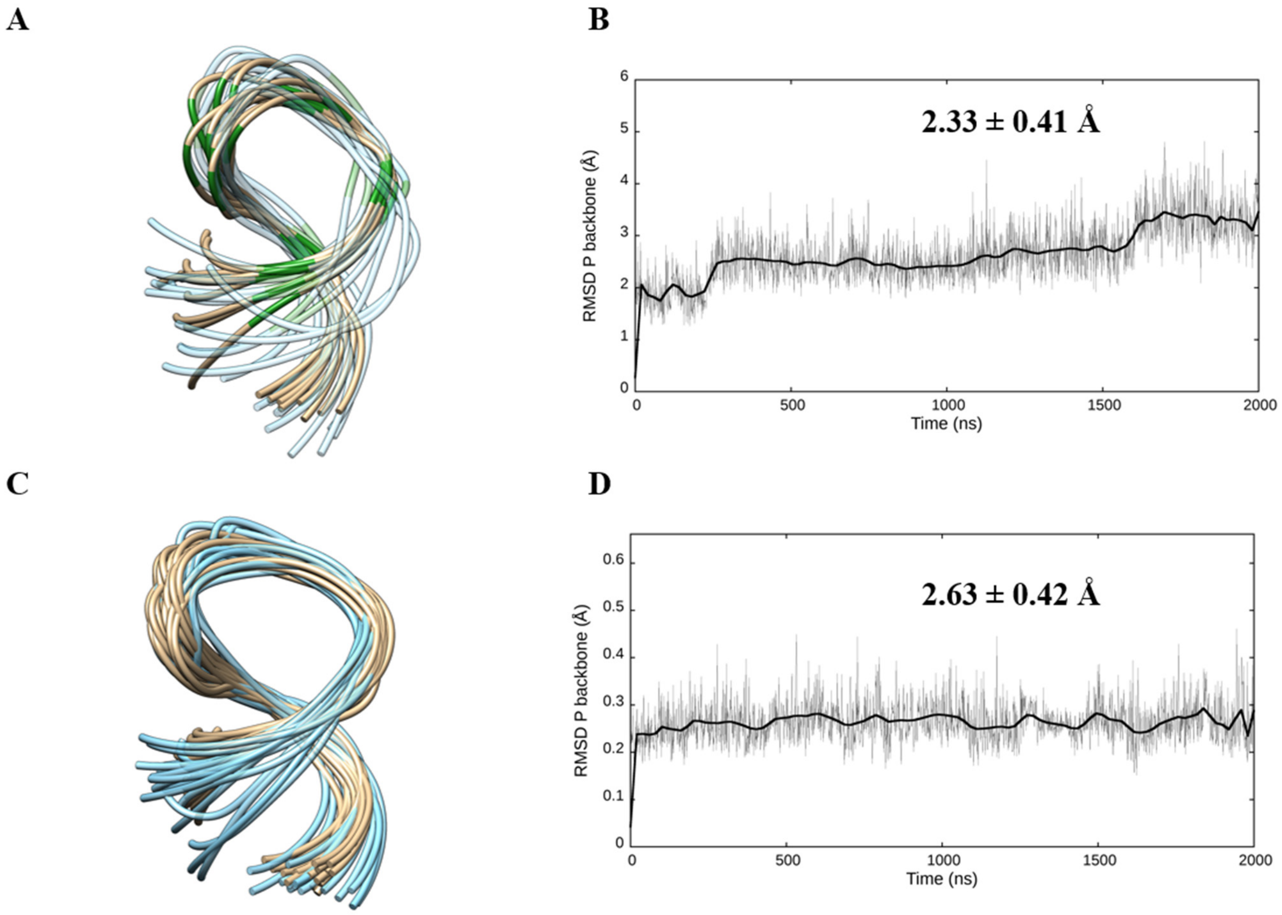

Figure S4: Structural data from the MD simulations. (A) Superposition of the most populated cluster during 2000 ns classical MD (blue), onto the NMR ensembles (tan) of phosphorothioate modified RNA binding site for phage MS2 coat protein. (B) RMSDs of phosphorous backbone atom during the 2000 ns Classical MD. The positions of the PSs are shown in green. (C) Superposition of the most populated clusters during 2000 ns Classical MD (blue), onto the NMR ensembles (tan) of natural RNA binding site for phage MS2 coat protein (PDB code: 1D0U). (D) RMSDs of P backbone atom during the 2000 ns Classical MD for the natural RNA(PDB code: 1D0U).

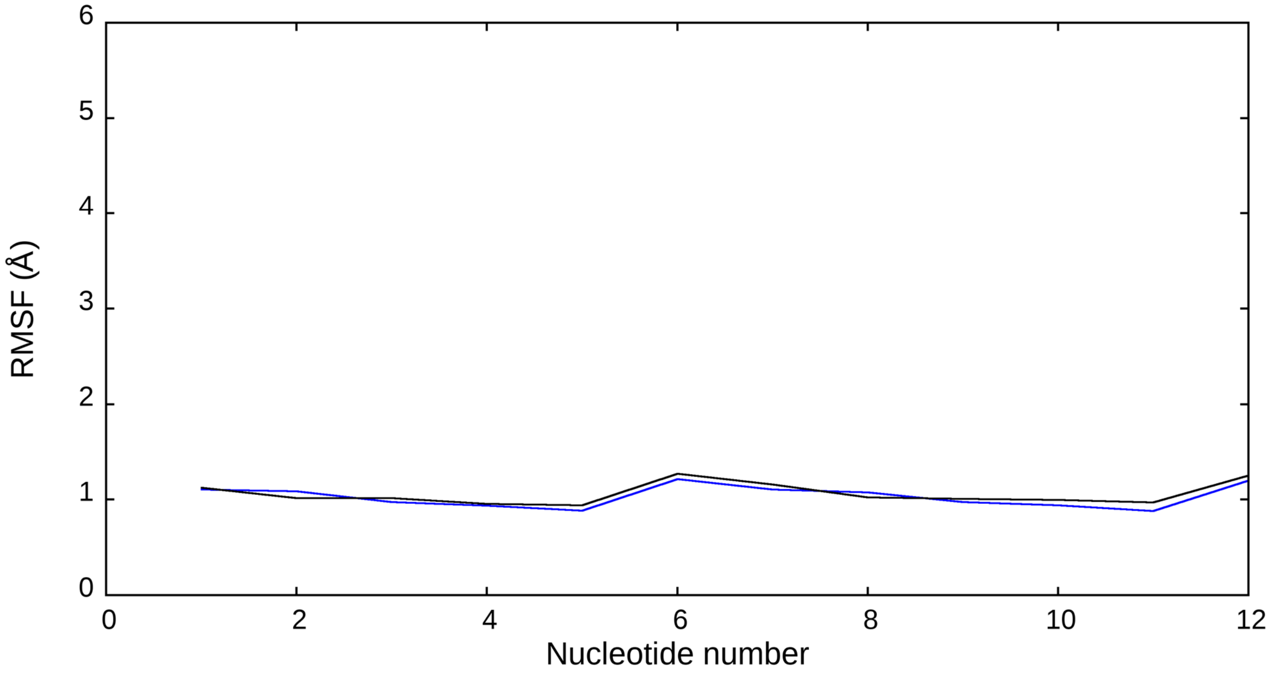

Figure S5. RMSF of residues along the PS (PDB code: 1D97) and natural-ONs, in blue and black, respectively.

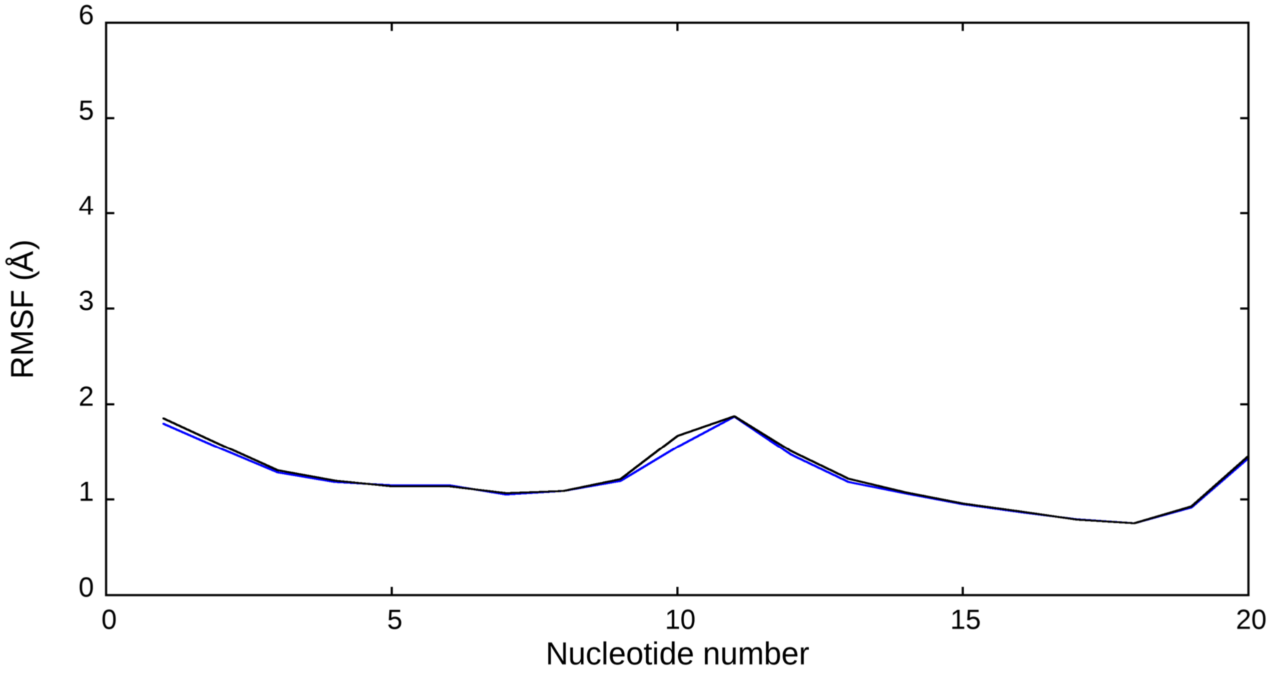

Figure S6. RMSF of residues along the PS (PDB code: 219D) and natural-ONs, in blue and black, respectively.

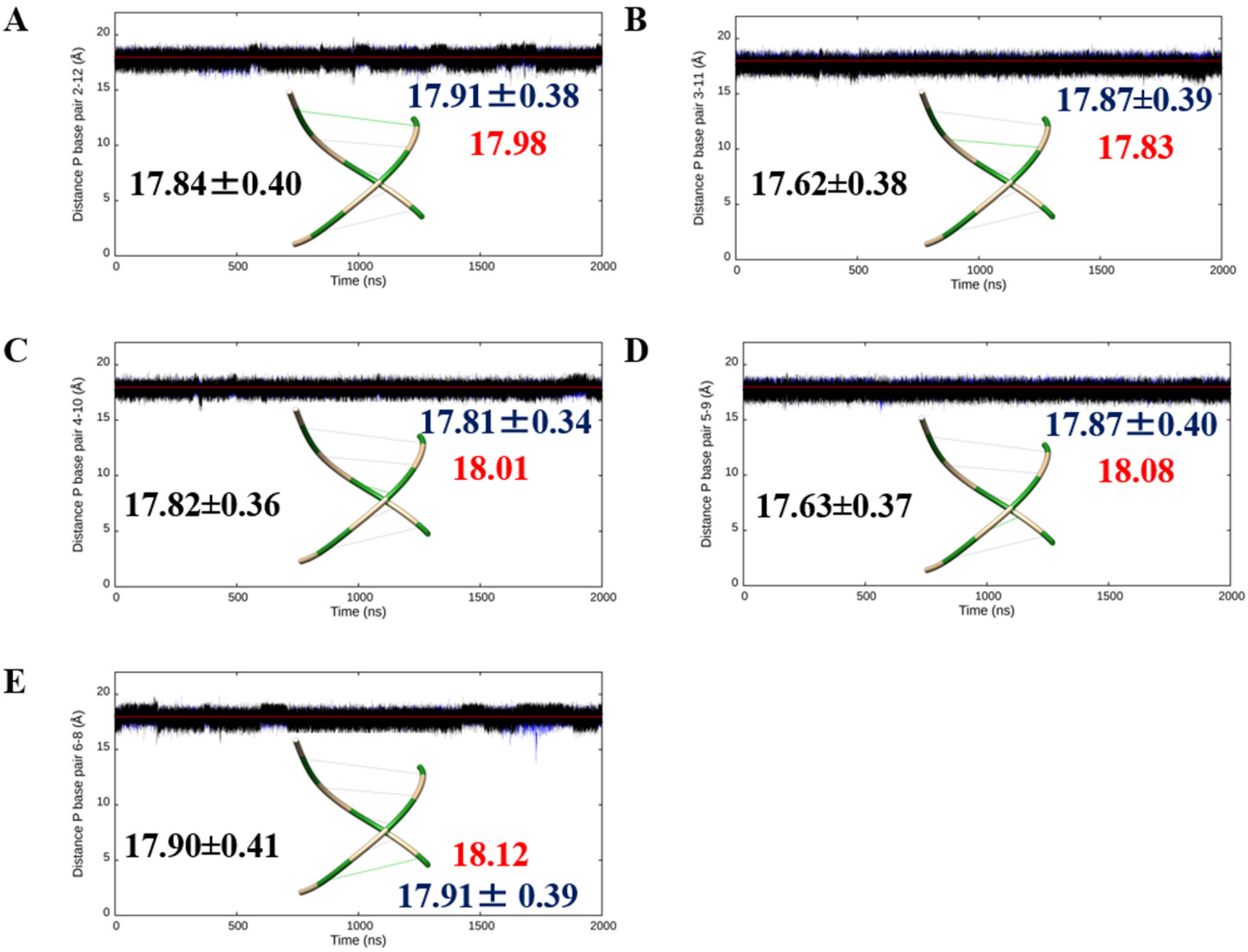

Figure S7. Inter-strand phosphate distances for each base pair in position (A) 2-12, (B) 3-11, (C) 4-10, (D) 5-9, (E) 6-8 colored in blue and black for PS and natural-ONs, respectively. Red lines and green dotted lines represent experimental inter-strand phosphate distances in PDB code 1D97 and representative inter-strand phosphate distances for each base pair, respectively. Average distance and standard deviations with experimental inter-strand P-P distances are reported in Å, blue for the PS B-DNA MD trajectory, red for the crystal structure, and black for natural B-DNA MD trajectory. The nucleotides with the phosphorothioate-modified backbone are shown in green.

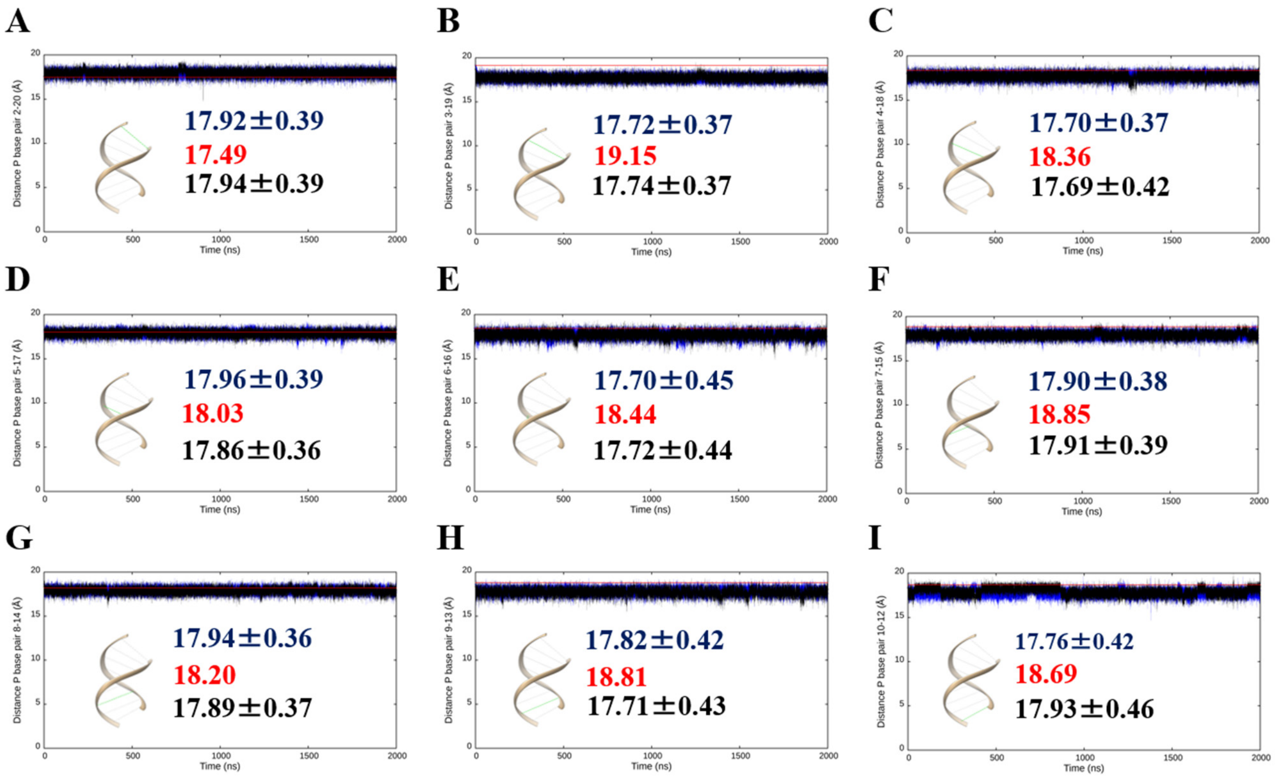

Figure S8: Inter-strand phosphate distance for each base pair in position (A) 2-20, (B) 3-19, (C) 4-18, (D) 5-17, (E) 6-16, (F) 7-15, (G) 8-14, (H) 9-13, and (I) 10-12 colored in blue and black for PS and natural-ONs, respectively. Red lines and green dotted lines represent experimental inter-strand phosphate distances in PDB code 219D and representative inter-strand phosphate distances for each base pair, respectively. Average distance plus STD with experimental inter-strand P-P distances are reported in Å, blue for PS trajectory, red for PS NMR structure, and black for natural-ON trajectory. Position of the PSs are shown in green.

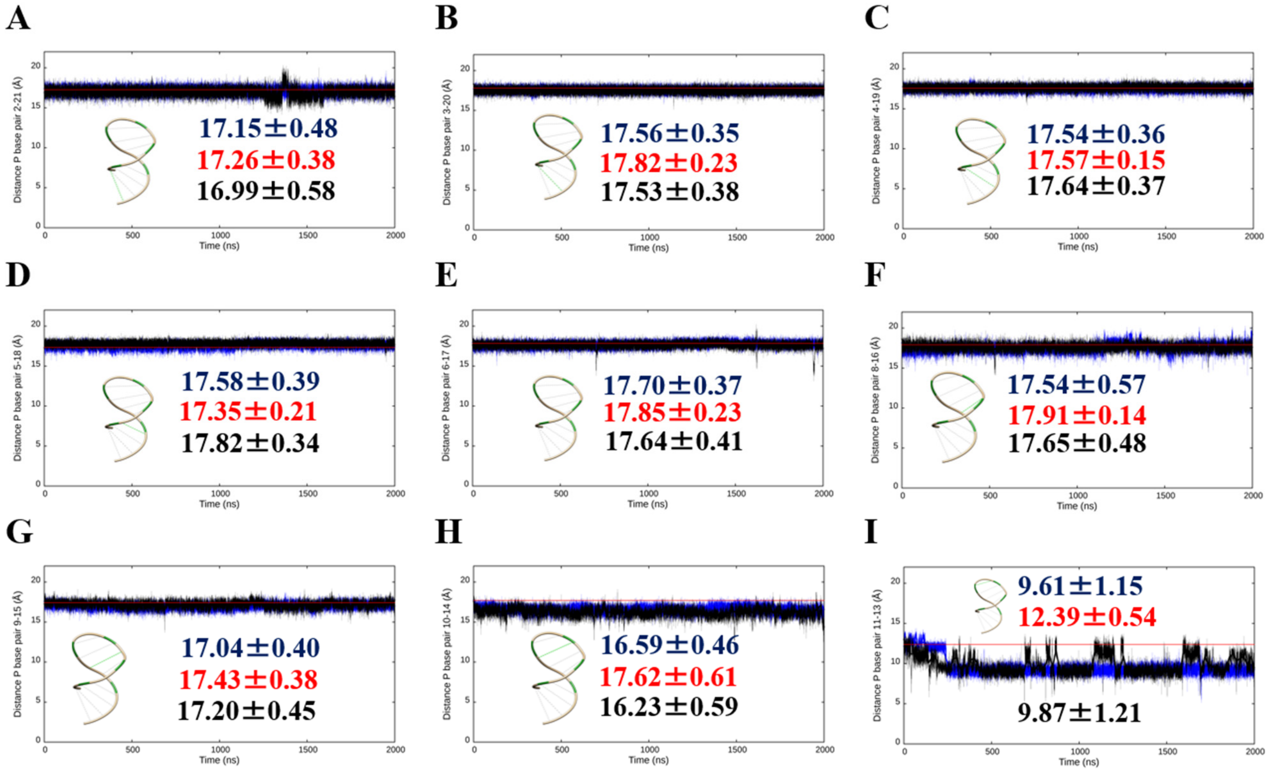

Figure S9: Inter-strand phosphate distance for each base pair in position (A) 2-21, (B) 3-20, (C) 4-19, (D) 5-18, (E) 6-17, (F) 8-16, (G) 9-15, (H) 10-12, and (I) 11-13 colored in blue and black for PS (PDB code: 1D0T) and natural-ONs (PDB code 1D0U), respectively. Red lines and green dotted lines represent experimental inter-strand phosphate distances in PDB code 1D0T and representative inter-strand phosphate distances for each base pair, respectively. Average distance plus STD with experimental inter-strand P-P distances are reported in Å, blue for PS trajectory, red for PS NMR ensemble, and black for natural-ODN trajectory. Position of the PSs are shown in green.

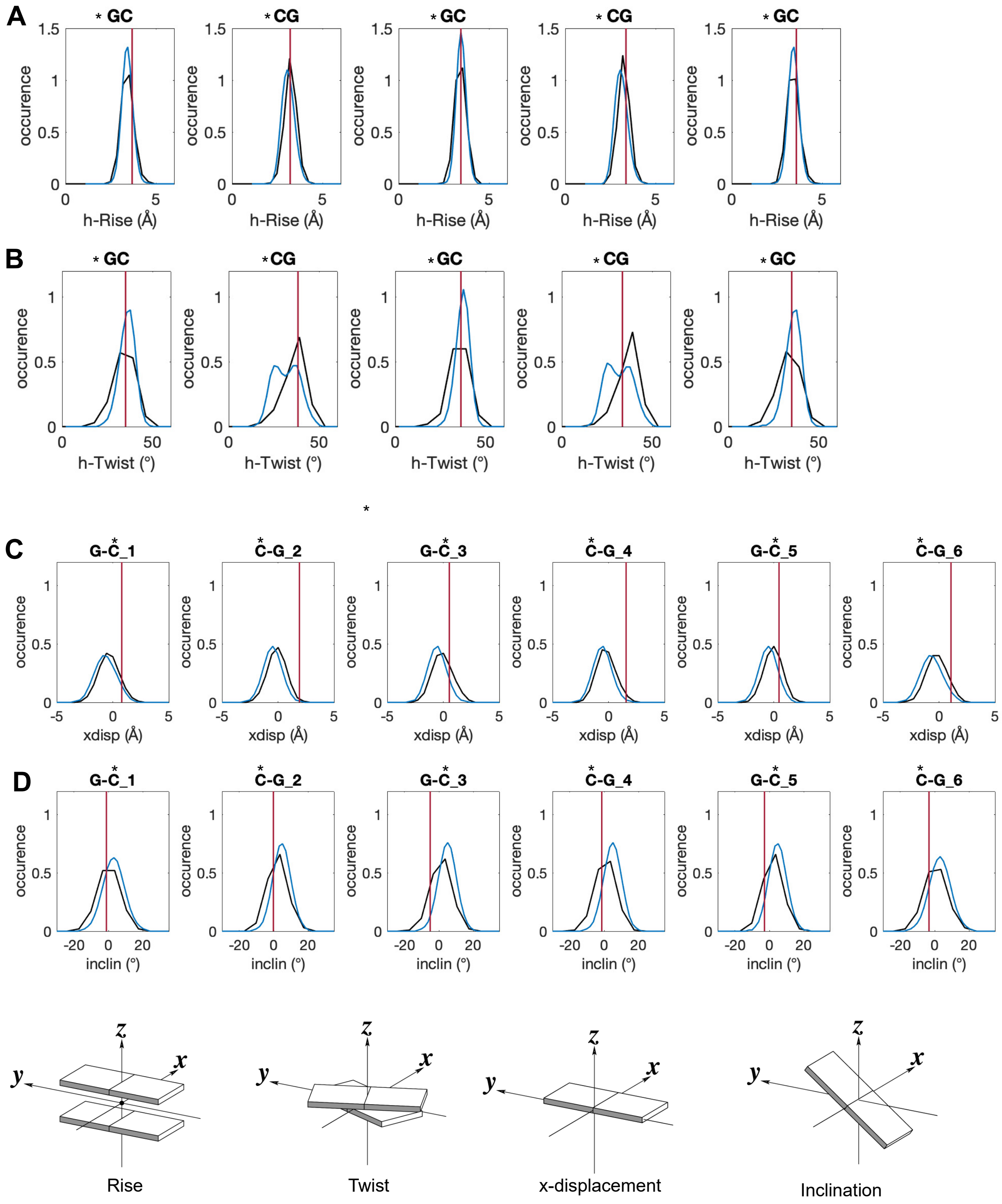

Figure S10: Helical parameters A) h-rise B) h-twist C) x-displacement D) inclination, for PS (blue) and natural-ONs (black), respectively. Red line illustrates the value for the x-ray structure "1d97". The location of the PS modifications is highlighted with "*".

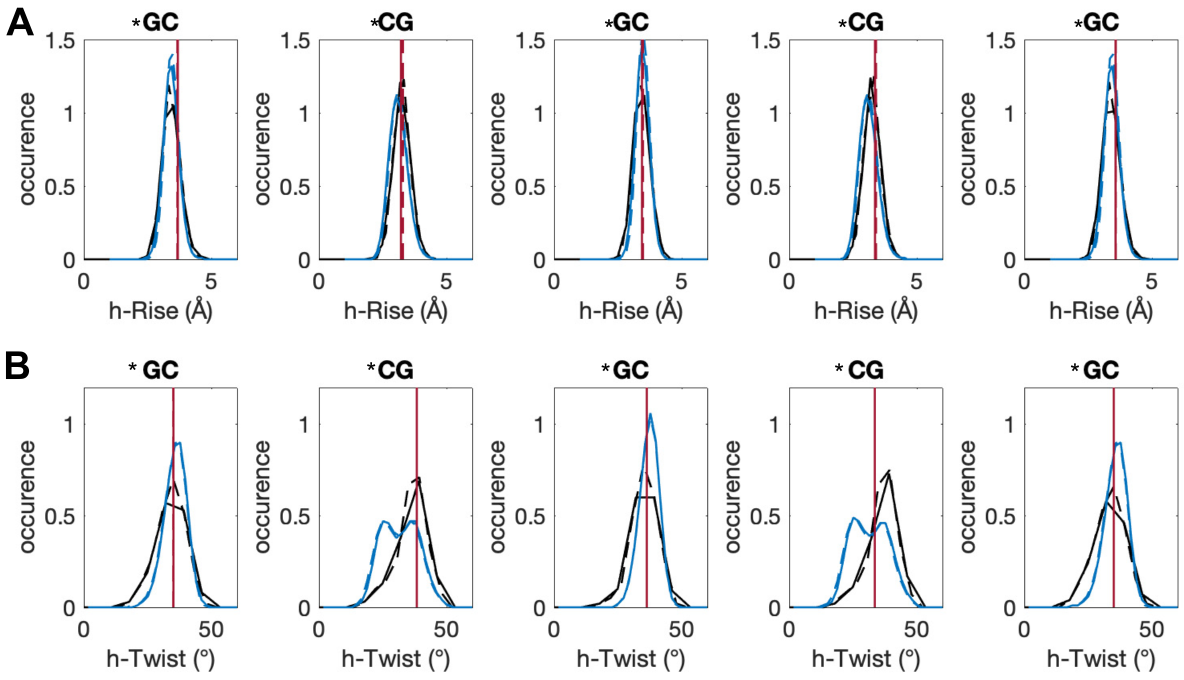

Figure S11: Comparison of **A.** h-rise (bold lines) and rise (dashed lines) and B. h-twist (bold lines) and twist (dashed lines). Large deviation is common for the A-form conformation. PS (blue) and natural-ONs (black), respectively. Red line illustrates the value for the x-ray structure "1d97". The location of the PS modifications is highlighted with "*".

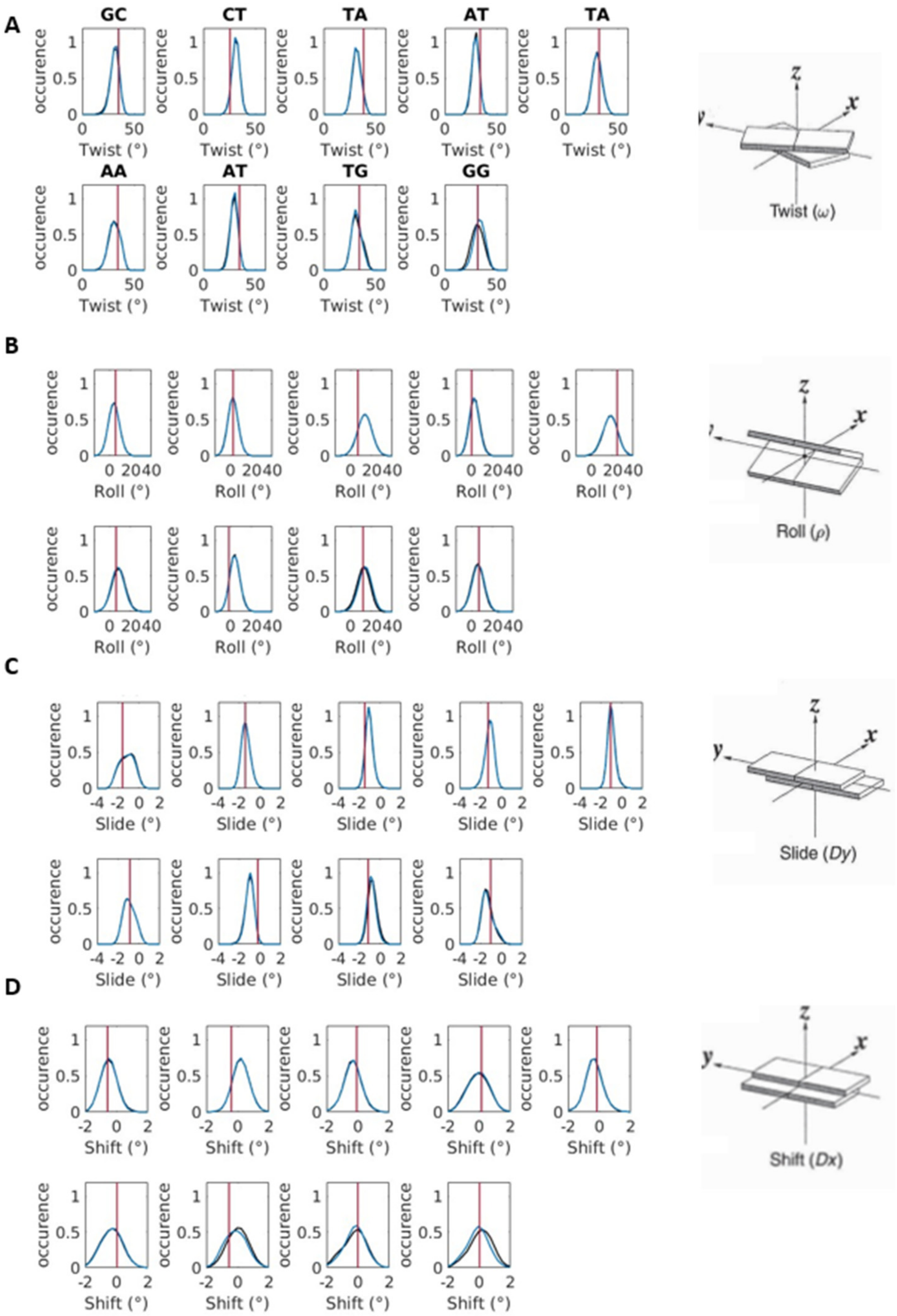

Figure S12. Distributions for (A) twist, (B) roll, (C) slide, and (D) shift for PS (blue) and natural-ONs (black), respectively. Red lines represent experimental helical parameters for each base pair step.

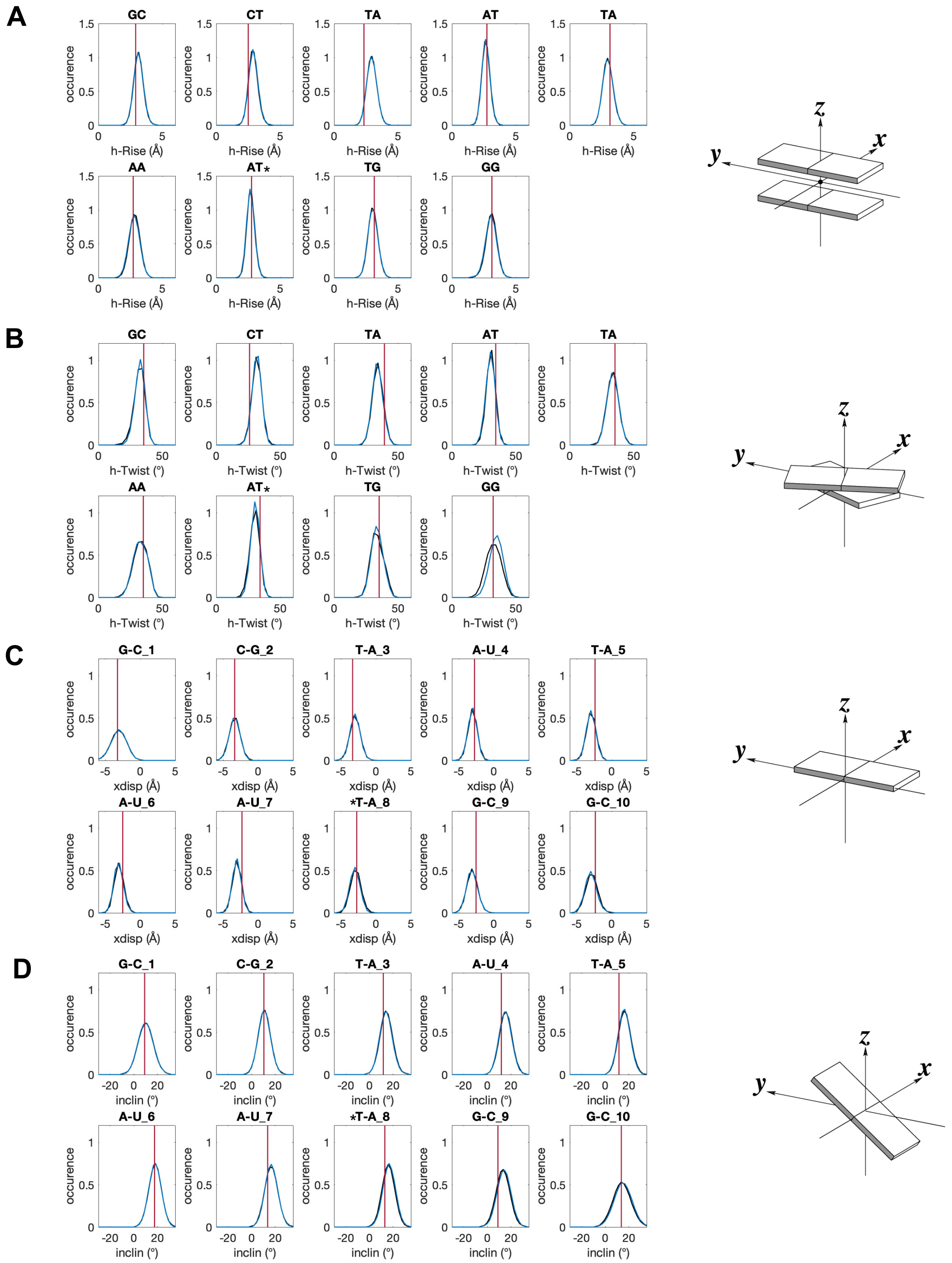

Figure S13: Helical parameters A) h-rise B) h-twist C) x-displacement D) inclination, for PS (blue) and natural-ONs (black), respectively. Red line illustrates the value for the nmr structure “219D”. The location of the PS modifications is highlighted with “*”.

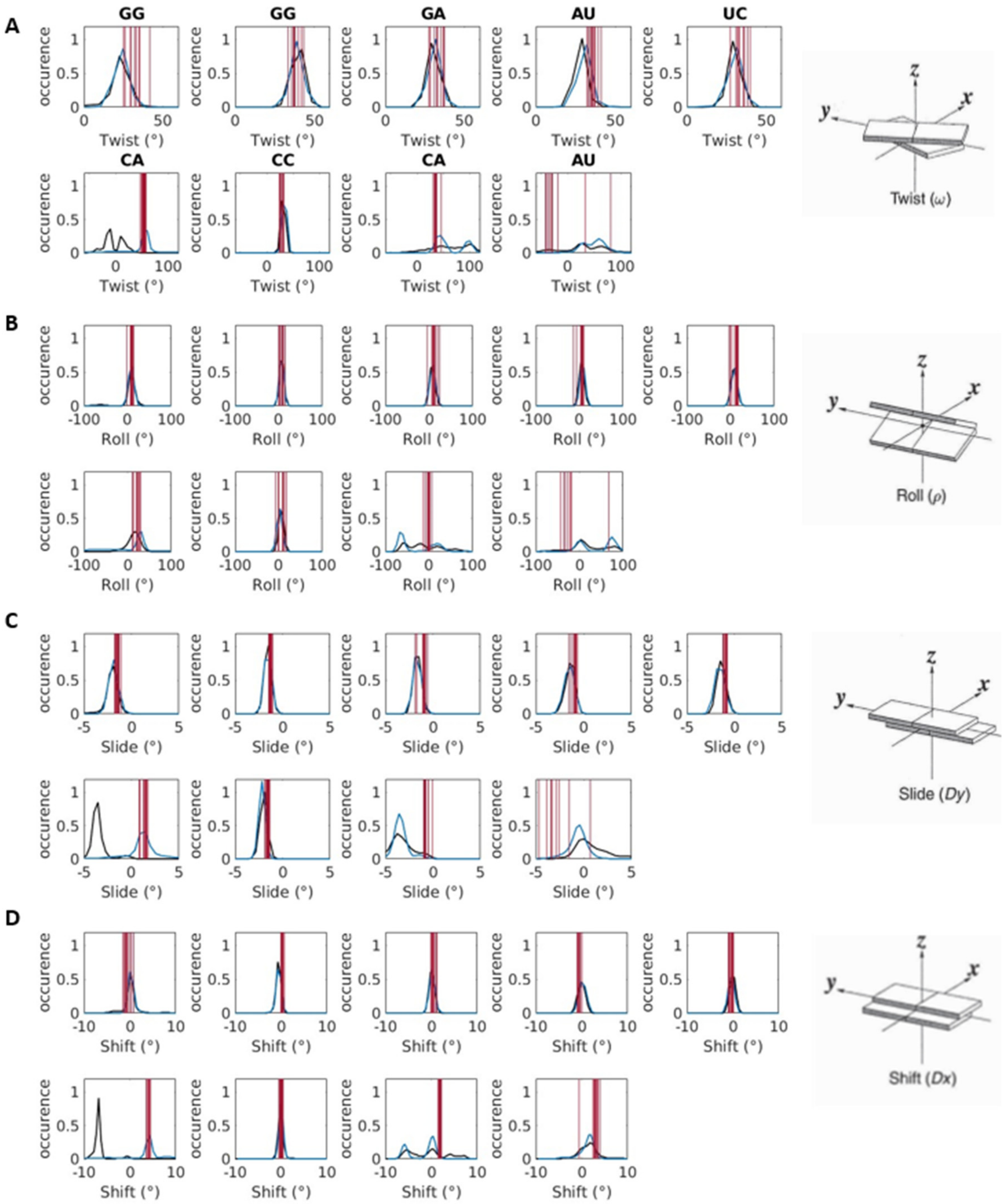

Figure S14. Distributions of (A) twist, (B) roll, (C) slide, and (D) shift for PS (blue) and natural-ONs (black), respectively. Red lines represent experimental helical parameters for each base pair step.

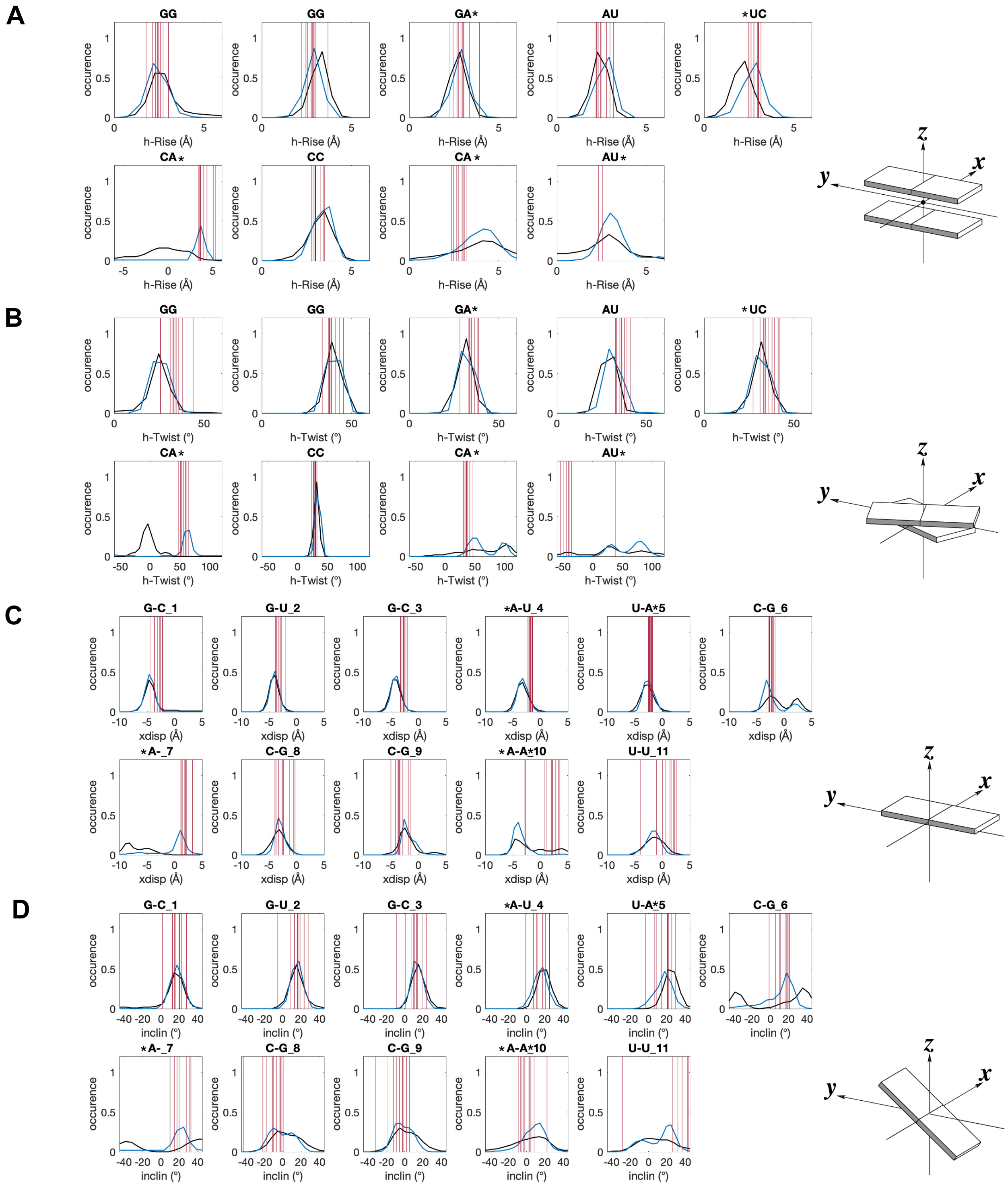

Figure S15: Helical parameters A) h-rise B) h-twist C) x-displacement D) inclination for PS (blue) and natural-ONs (black), respectively. Red line illustrates the value for the nmr structure “1d0t”. The location of the PS modifications is highlighted with “*”.

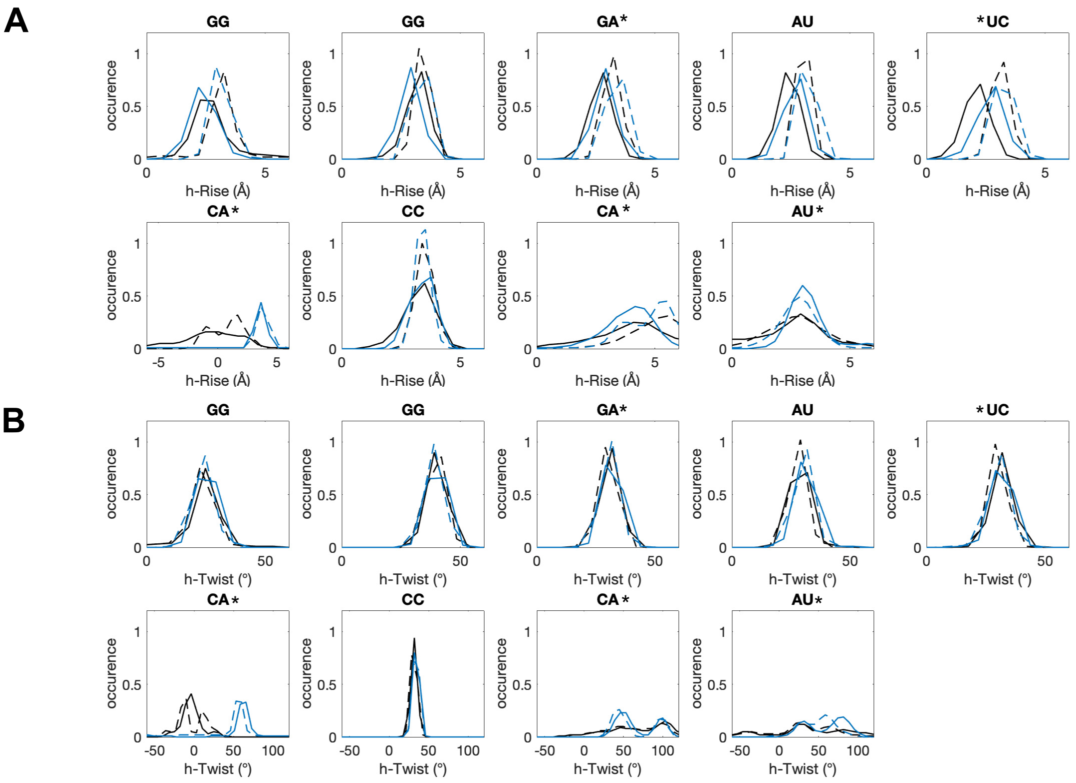

Figure S16: Comparison of **A.** h-rise (bold lines) and rise (dashed lines) and **B**. h-twist (bold lines) and twist (dashed lines). Large deviation is common for the A-form conformation. PS (blue) and natural-ONs (black), respectively. The location of the PS modifications is highlighted with "*". Red lines for the nmr data are excluded to make the comparison more visible.

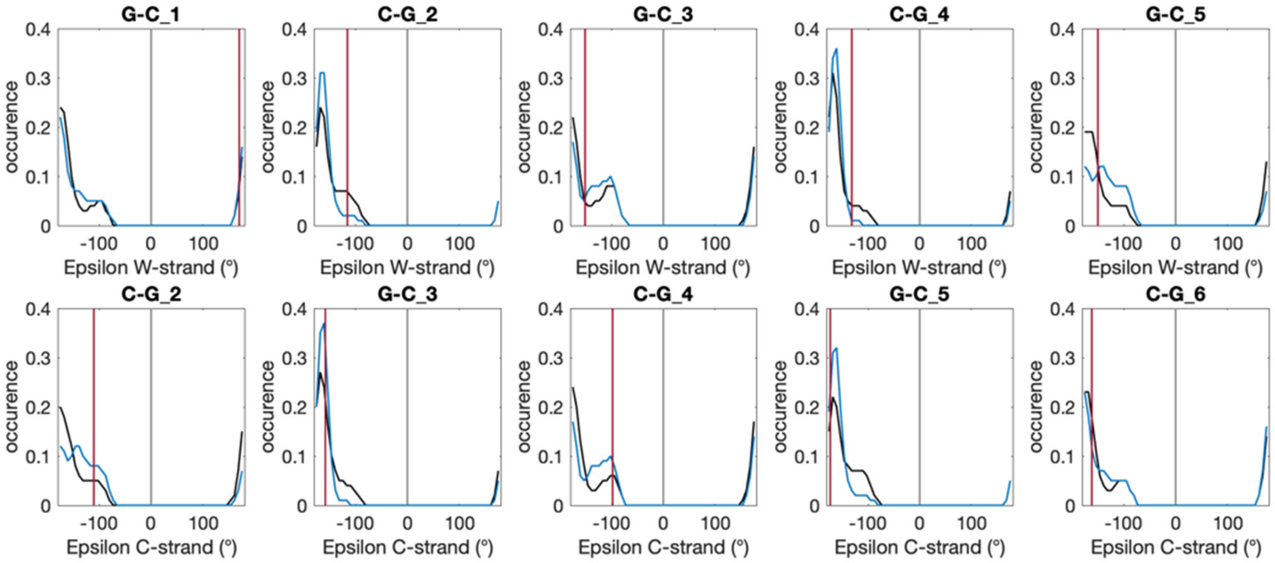

Figure S17. Most populated ε torsional angle in W-strand and C-strand for PS (blue) and natural-ONs (black), respectively. Red line illustrates the value for the x-ray structure “1d97”

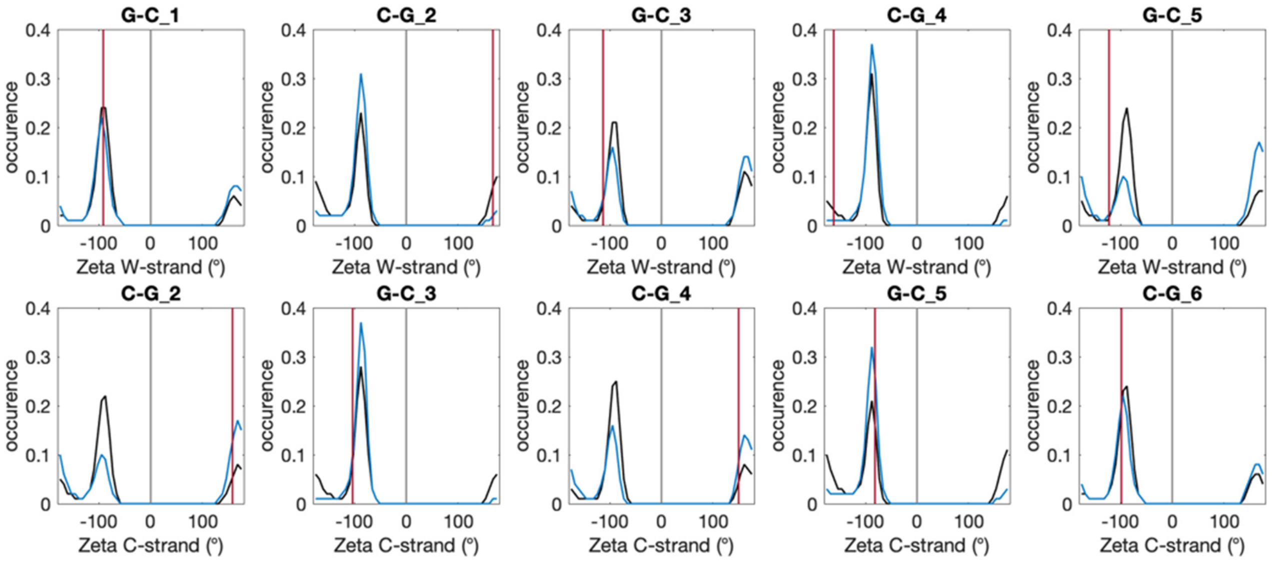

Figure S18. Most populated ζ torsional angle in W-strand and C-strand for PS (blue) and natural-ONs (black), respectively. Red line illustrates the value for the x-ray structure “1d97”

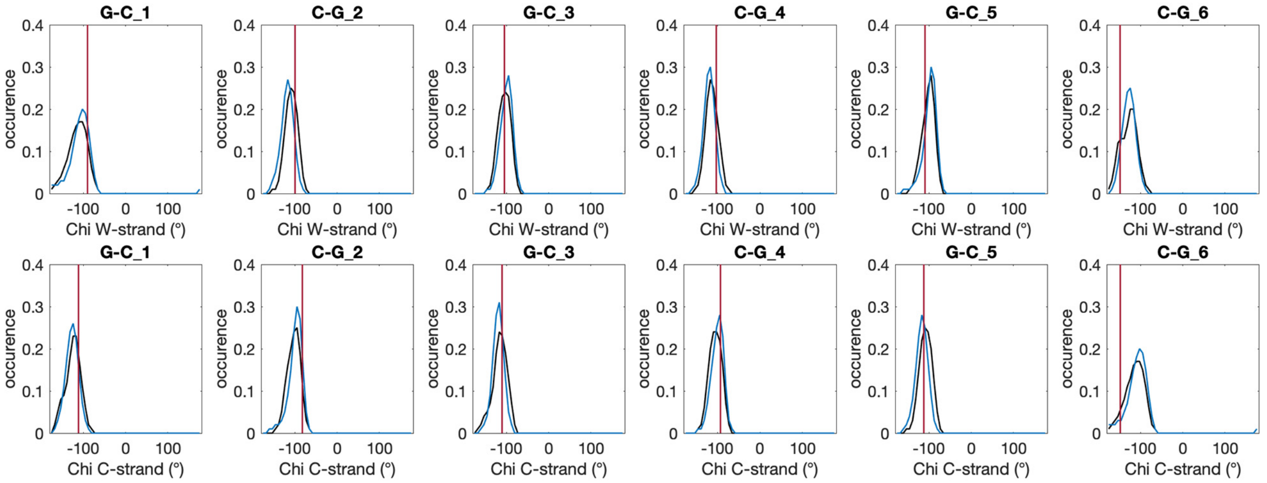

Figure S19: Most populated χ torsional angle in W-strand and C-strand for PS (blue) and natural-ONs (black), respectively. Red line illustrates the value for the x-ray structure "1d97"

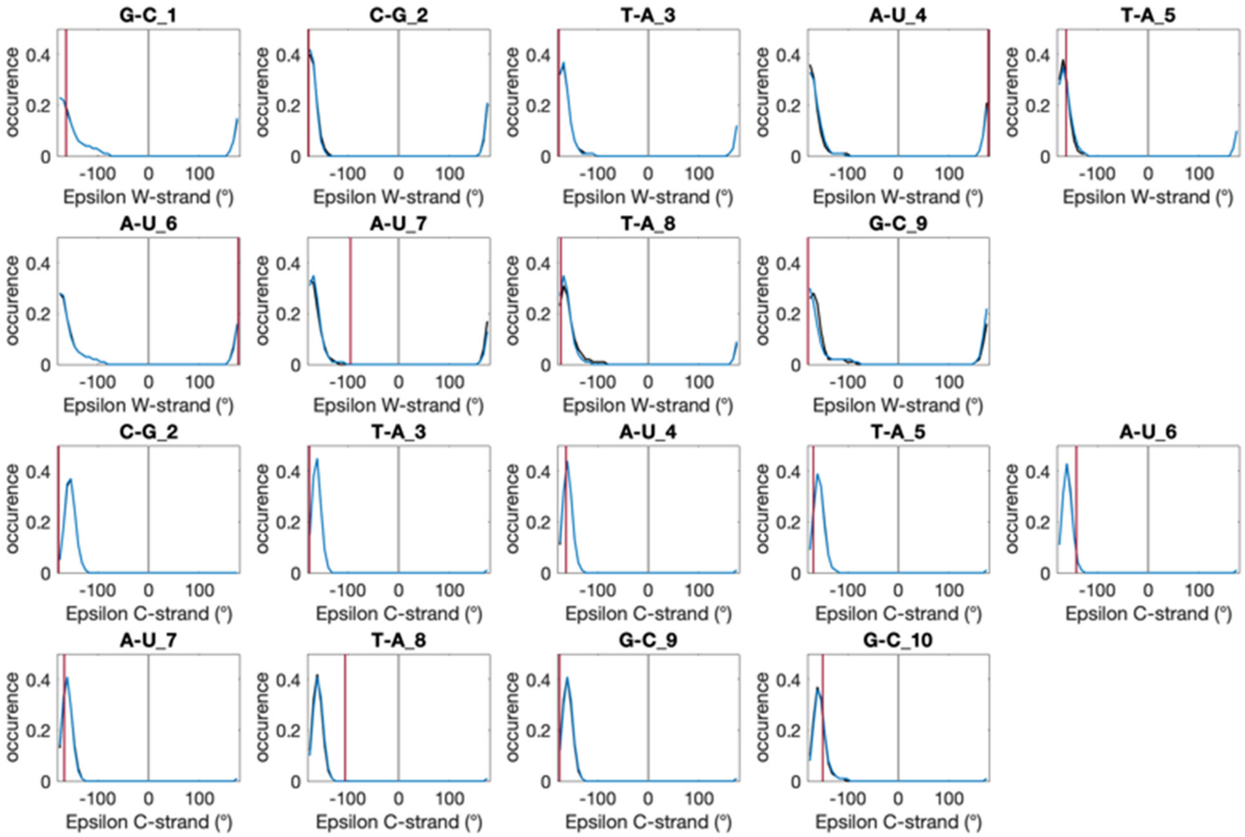

Figure S20. Most populated ε torsional angle in W-strand and C-strand for PS (blue) and natural-ONs (black), respectively. Red line illustrates the value for the nmr structure "219D"

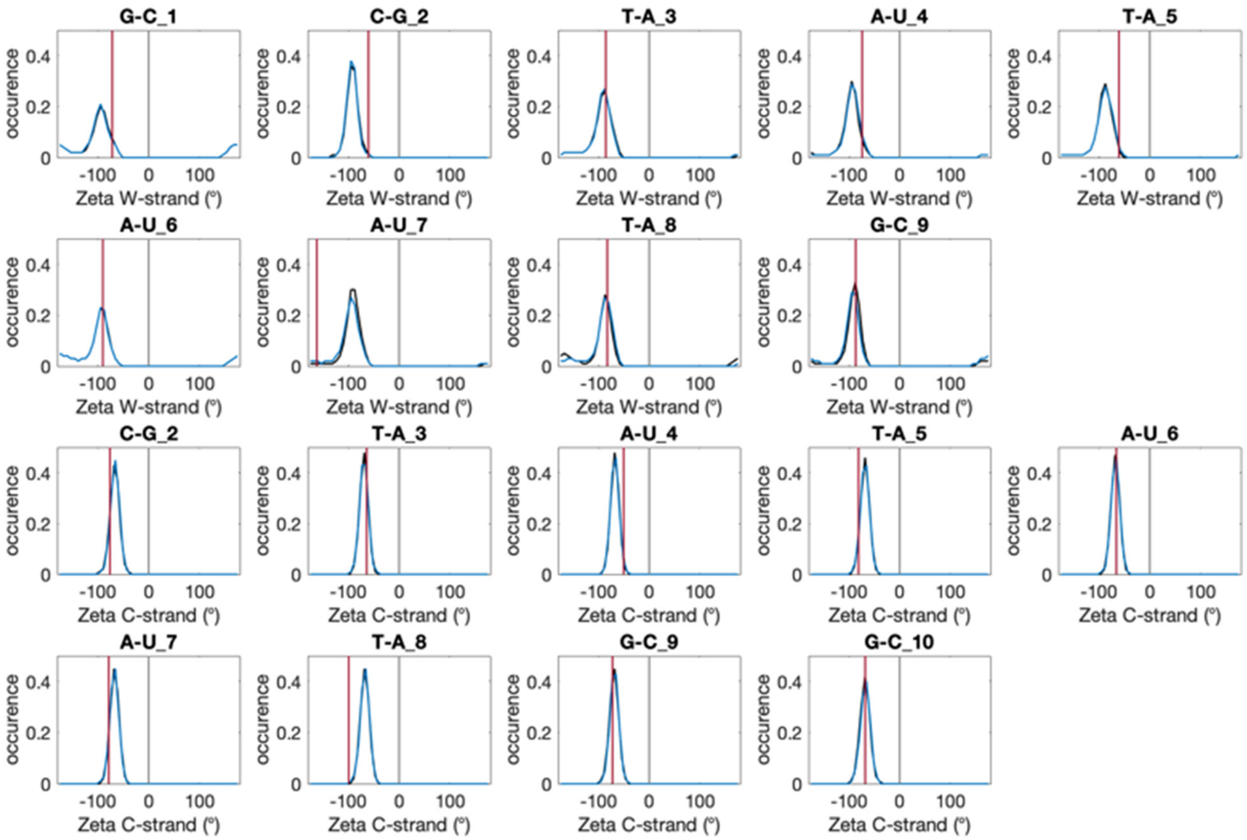

Figure S21. Most populated ζ torsional angle in W-strand and C-strand for PS (blue) and natural-ONs (black), respectively. Red line illustrates the value for the nmr structure "219D"

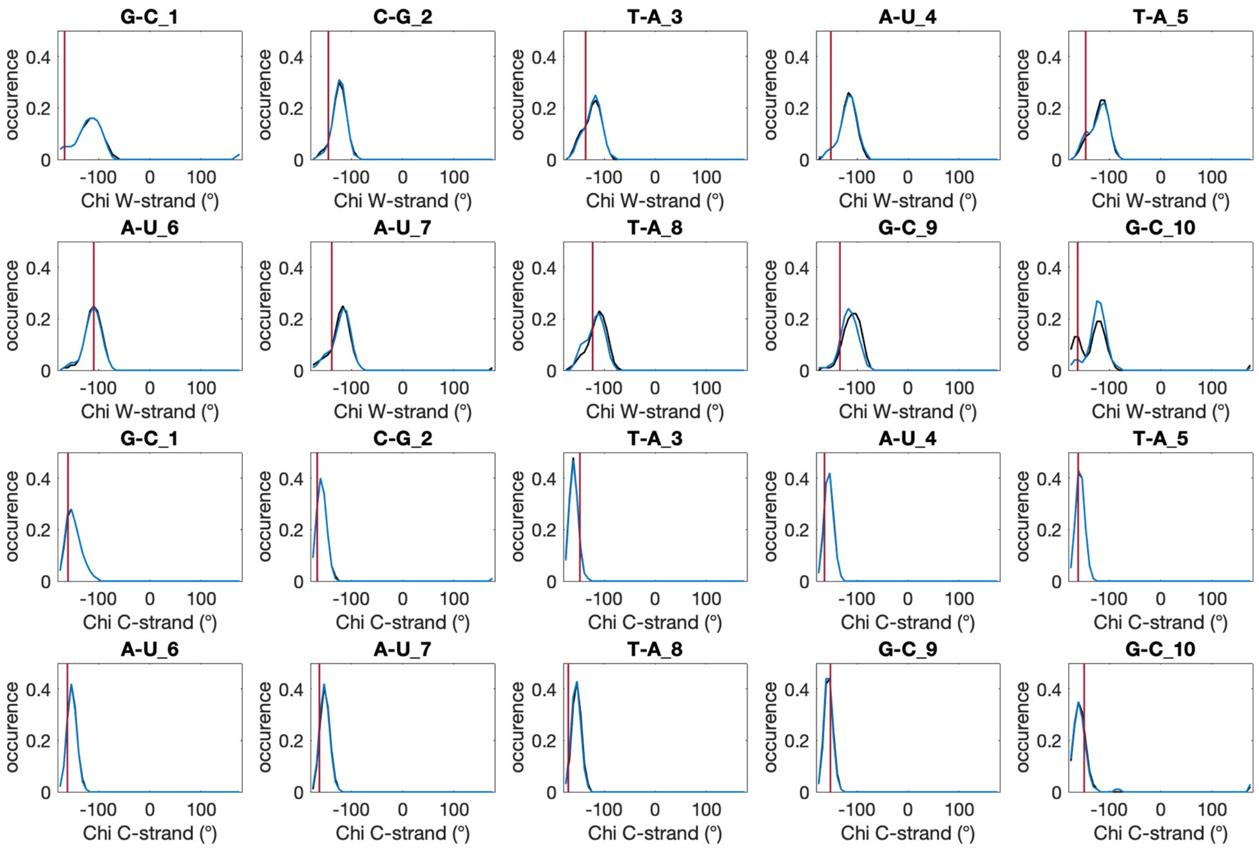

Figure S22: Most populated χ torsional angle in W-strand and C-strand for PS (blue) and natural-ONs (black), respectively. Red line illustrates the value for the nmr structure "219D"

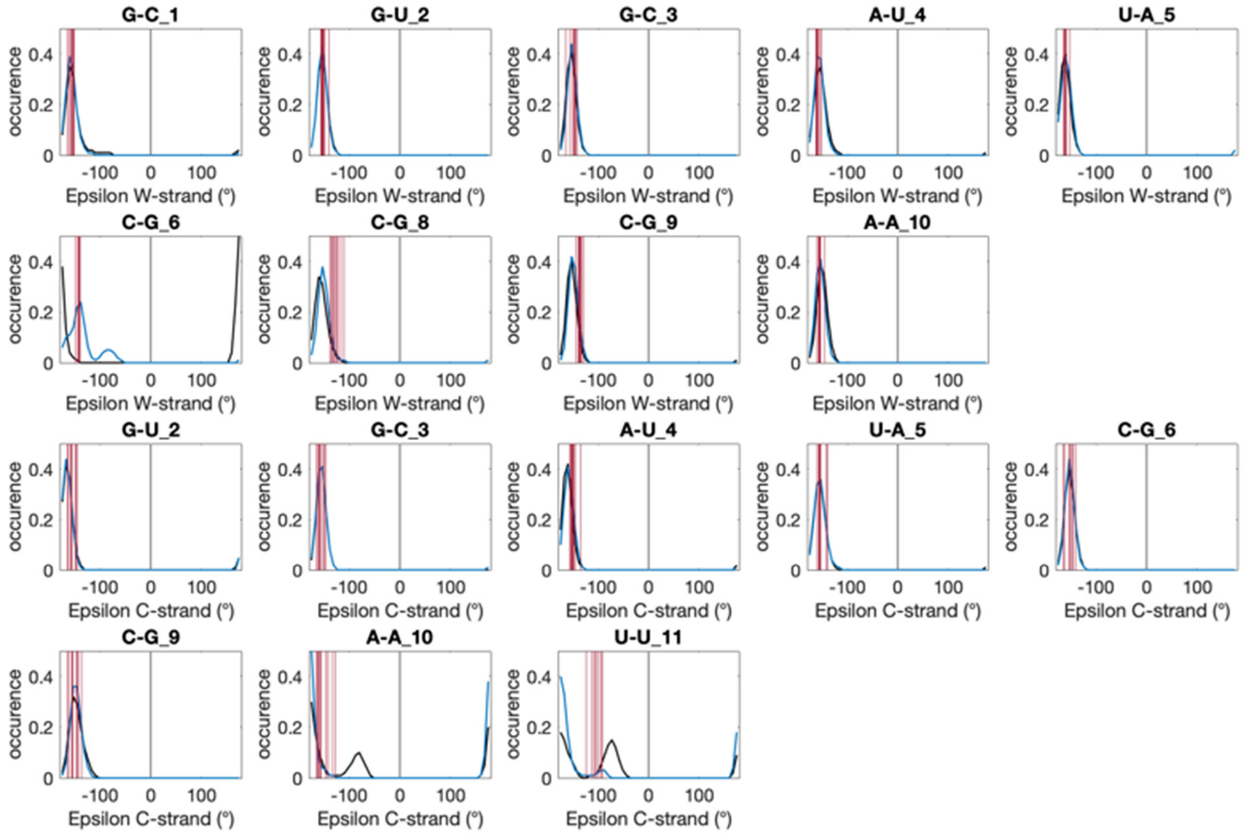

Figure S23. Most populated ε torsional angle in W-strand and C-strand for PS (blue) and natural-ONs (black), respectively. Red line illustrates the value for the nmr structure "1d0t"

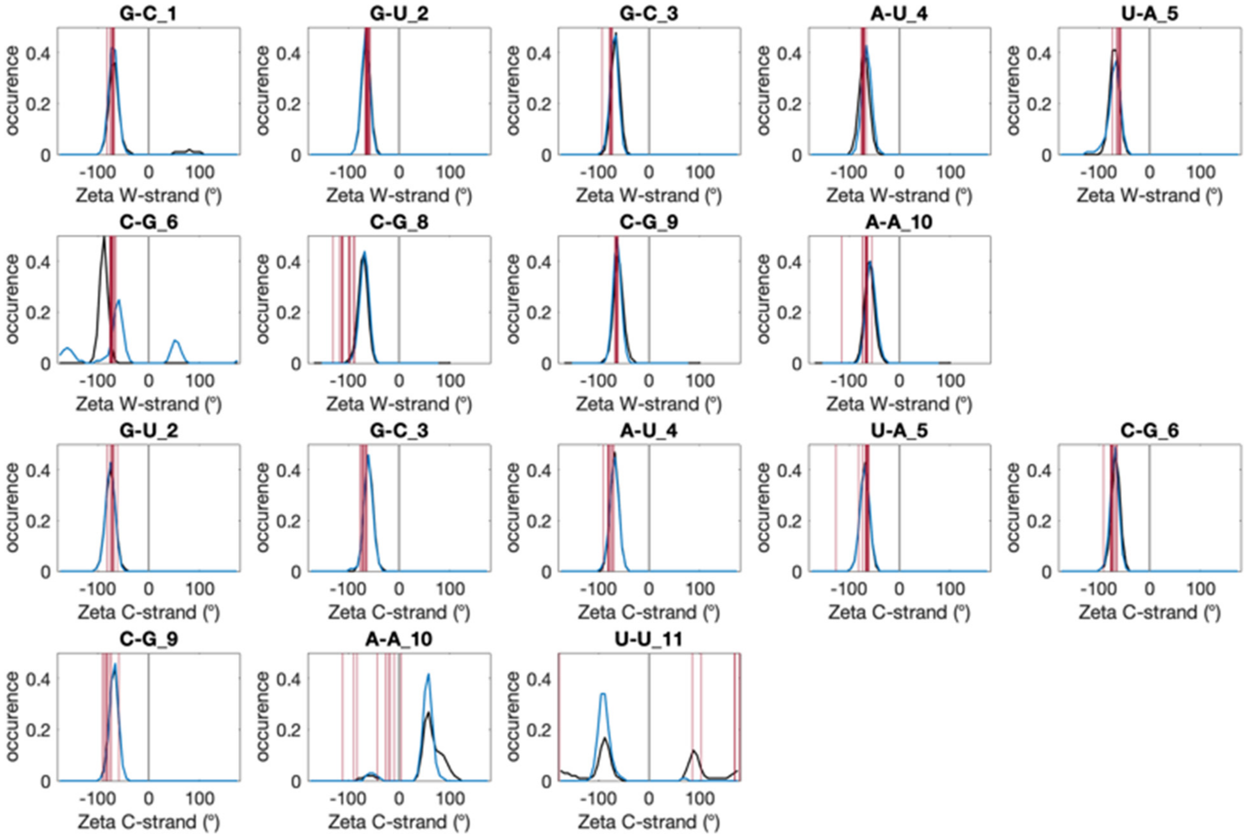

Figure S24. Most populated ζ torsional angle in W-strand and C-strand for PS (blue) and natural-ONs (black), respectively. Red line illustrates the value for the nmr structure "1d0t"

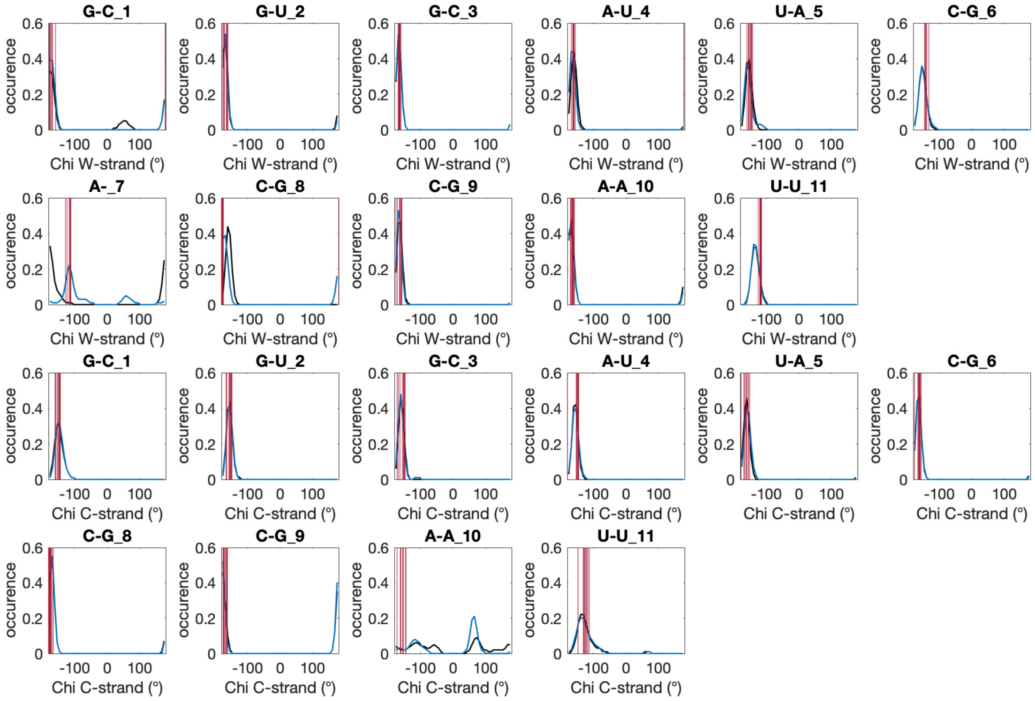

Figure S25. Most populated χ torsional angle in W-strand and C-strand for PS (blue) and natural-ONs (black), respectively. Red line illustrates the value for the nmr structure "1d0t"

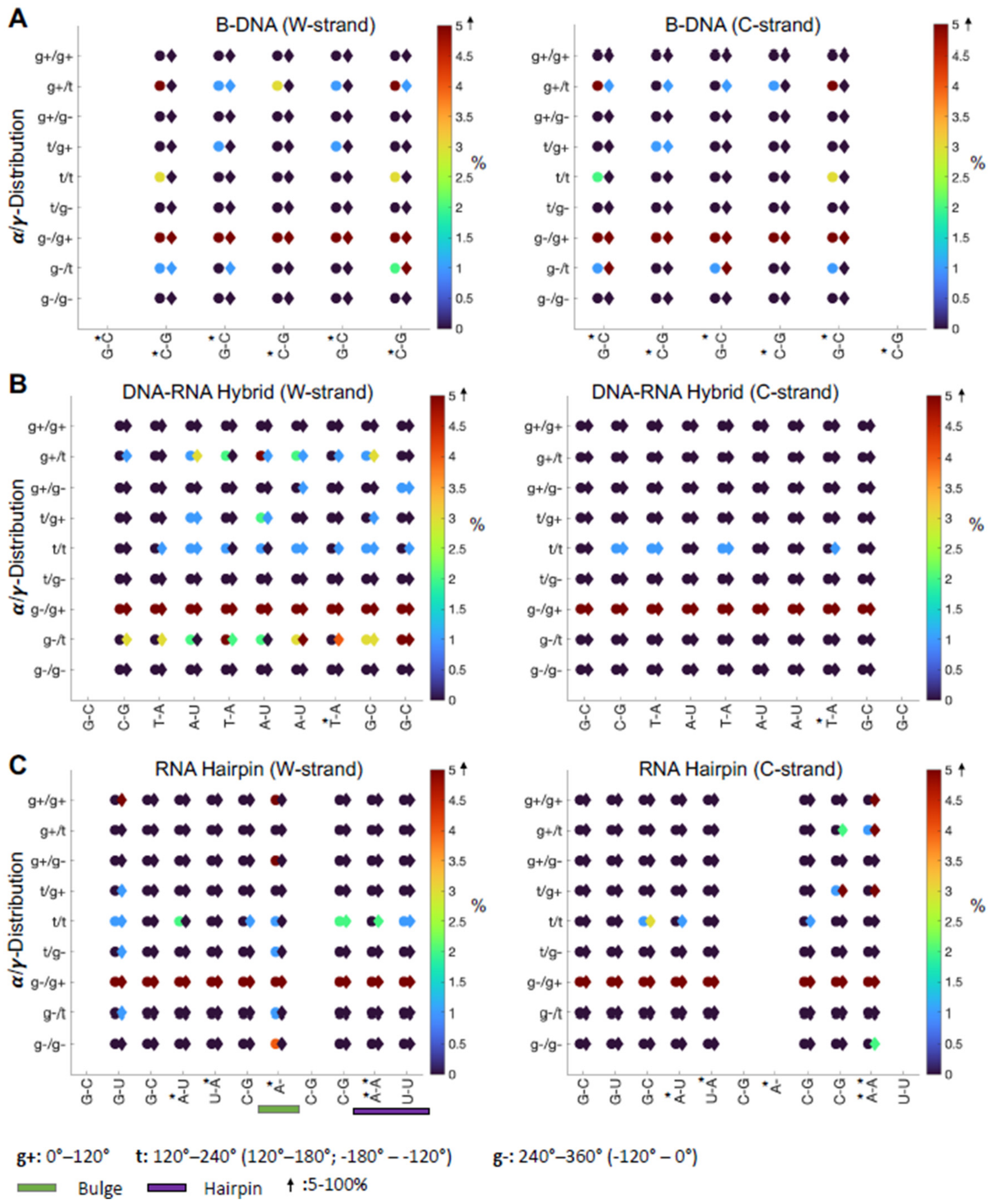

Figure S26. α/γ-distributions (%) for **A.** B-DNA **B.** DNA:RNA hybrid duplex **C.** Hairpin-RNA. PS modified systems are denoted with colored circles and Natural systems are denoted with colored diamonds. The location of the PS modifications is highlighted with "*".

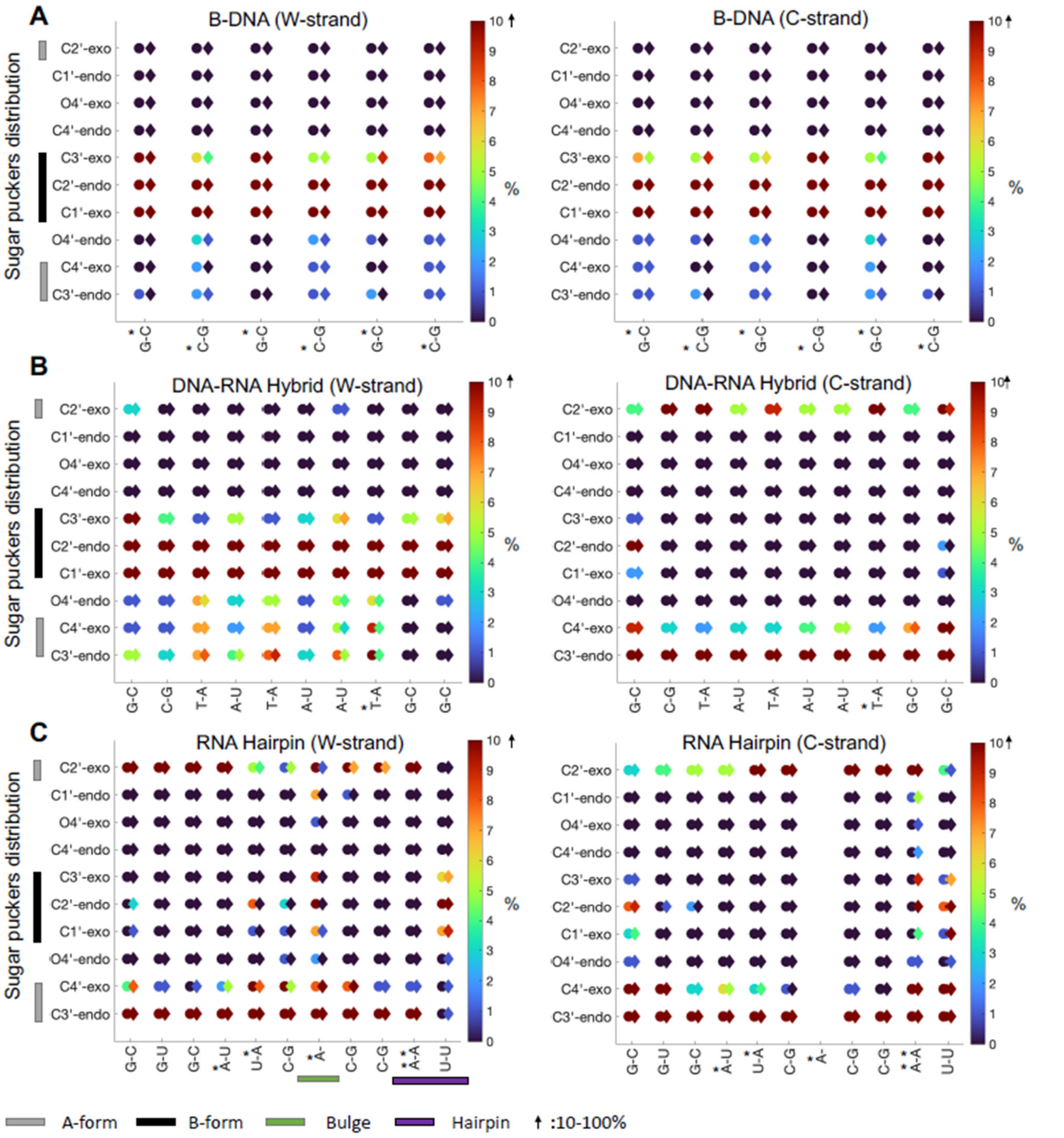

Figure S27- Sugar puckers distributions (%) for **A.** B-DNA **B.** DNA:RNA hybrid duplex **C.** Hairpin-RNA. PS modified systems are denoted with colored circles and Natural systems are denoted with colored diamonds. The location of the PS modifications is highlighted with “*”.

Table S1. Partial charges assigned to the phosphorothioate and the neighboring carbon atoms.

Atom name	Atom type	Partial charge (e)<br>This work	Partial charge (e)<br>Genna *et. al*[1]
P	P	0.93000	0.6258
S1P	S2	-0.70000	-0.6100
O2P	O2	-0.60000	-0.6700
O5’	OS	-0.30000	-0.1816
C5	CI	0.05580	-0.006900
O3’	OS	-0.57000	-0.523200
C3’	CT	0.20220	0.071300

Table S2. P-S bond parameters assigned to the phosphorothioate.

Bond	r_{eq} (Å) and k (kcal/mol)<br><br>This work	r_{eq} (Å) and k (kcal/mol)<br><br>Genna *et. al*[1]
P-S2	1.998 / 243.750	2.016 / 525.0

Table S3. Parameters for the angles involving the newly introduced sulfur atom (S2).

Angle	$Angle_{eq}$ (°) and k (kcal/mol)<br><br>This work	$Angle_{eq}$ (°) and k (kcal/mol)<br><br>Genna *et. al*[1]
S2-P-O2	121.171 / 70.047	108.23 / 100
S2-P-OS	110.965 / 62.506	108.23 / 100

Table S4. Summary of PS parameterization philosophies described in the main text.

	**Model**	**Approach**	**Parameters optimized**	**Validation system**	**Note**
Lind *et. al*[2]	DMPT	ab initio calculations + MM fitting	Atomic partial charges + LJ + torsional coefficient[a]	hybrid DNA:RNA duplexes	Parameter values not presented
Zhang *et al*[3]	DMPT	QM/MM calculations	Atomic partial charges[b]	RNA-ribostamycin complex	Parameter values not presented
Genna *et al*[1]	Dinucleotide	QM/MM well-tempered metadynamics	α/ζ angles	DNA:RNA hybrids duplexes	
Our study	DMPT	QM + MM optimization	Atomic partial charges + bond + angles + torsional	DNA, RNA, hybrid DNA:RNA duplex	Full parameter set available online

[a] atomic partial charges assigned using the RESP approach, Lennard-Jones (LJ) and torsional parameters by QM single points energy optimization to MM level of theory, while bond stretching and angle bending parameters were transferred without optimization from the AMBER 4.1 force field.

[b] atomic partial charges assigned using the RESP approach; other parameters assigned through a QM/MM approach, and assigning LJ parameters for the PS sulfur atom by analogy from CHARMM.

References

1. Genna, V., Iglesias-Fernandez, J., Reyes-Fraile, L., Villegas, N., Guckian, K., Seth, P., Wan, B., Cabrero, C., Terrazas, M., Brun-Heath, I. et al. (2023) Controlled sulfur-based engineering confers mouldability to phosphorothioate antisense oligonucleotides. *Nucleic Acids Res*, **51**, 4713-4725.
2. Lind, K. E., Sherlin, L. D., Mohan, V., Griffey, R. H., Ferguson, D. M. (1998) Parameterization and simulation of the physical properties of phosphorothioate nucleic acids. *Molecular Modeling of Nucleic Acids*, 41-54.
3. Zhang, Z., Vogele, J., Mrazikova, K., Kruse, H., Cang, X., Wohnert, J., Krepl, M. and Sponer, J. (2021) Phosphorothioate Substitutions in RNA Structure Studied by Molecular Dynamics Simulations, QM/MM Calculations, and NMR Experiments. *J Phys Chem B*, 125, 825-840.
