# Supplementary material for: Structural dynamics of therapeutic nucleic acids with phosphorothioate backbone modifications
Source: NAR Genom Bioinform. 2024 May 25;6(2):lqae058. doi: 10.1093/nargab/lqae058 (PMC11127634; doi:10.1093/nargab/lqae058)
Supplement: lqae058_Supplemental_Files [file lqae058_supplemental_files.zip › Video_description.docx]

**Video S1.** B-DNA structural stability during the 2 μs MD trajectory

**Video S2.** DNA:RNA duplex structural stability during the 2 μs MD trajectory

**Video S3.** ssRNA structural stability during the 2 μs MD trajectory

**Video S4.** A_7_ nucleotide behavior in natural ssRNA

**Video S5.** A_7_ nucleotide behavior in modified PS ssRNA

**Video S6.** Independent A10-U11 and U12-A13 base stacking events for modified PS ssRNA

**Video S7.** Independent A10-U11 and U12-A13 base stacking events for natural ssRNA
